# Comparative oesophageal cancer risk assessment of hot beverage consumption (coffee, mate and tea): the margin of exposure of PAH vs very hot temperatures

**DOI:** 10.1186/s12885-018-4060-z

**Published:** 2018-03-01

**Authors:** Alex O. Okaru, Anke Rullmann, Adriana Farah, Elvira Gonzalez de Mejia, Mariana C. Stern, Dirk W. Lachenmeier

**Affiliations:** 1 Chemisches und Veterinäruntersuchungsamt (CVUA) Karlsruhe, Weissenburger Strasse 3, D-76187 Karlsruhe, Germany; 2 Department of Pharmaceutical Chemistry, University of Nairobi, Off Ngong Road, P.O. Box 19676-00202, Nairobi, Kenya; 3 Nutrition Institute, Centre of Health Sciences, Federal University of Rio de Janeiro, Bloco J - Av. Carlos Chagas Filho 373, Ilha do Fundão, Rio de Janeiro, Brazil; 4 Department of Food Science and Human Nutrition, Division of Nutritional Sciences, University of Illinois, 228 ERML, 1201 W. Gregory Drive, Urbana, IL 61801 USA; 5 Department of Preventive Medicine, Norris Comprehensive Cancer Center, Keck School of Medicine of USC, Los Angeles, CA USA

**Keywords:** Coffee, Tea, Mate, Polycyclic aromatic hydrocarbons, Hot temperature, Beverages, Esophageal cancer

## Abstract

**Background:**

Consumption of very hot (> 65 °C) beverages is probably associated with increased risk of oesophageal cancer. First associations were reported for yerba mate and it was initially believed that high content of polycyclic aromatic hydrocarbons (PAH) might explain the risk. Later research on other beverage groups such as tea and coffee, which are also consumed very hot, found associations with increased risk of oesophageal cancer as well. The risk may therefore not be inherent in any compound contained in mate, but due to temperature. The aim of this study was to quantitatively assess the risk of PAH in comparison with the risk of the temperature effect using the margin of exposure (MOE) methodology.

**Methods:**

The human dietary benzo[*a*]pyrene (BaP) and PAH4 (sum of benzo[*a*]pyrene, benzo[*a*]anthracene, chrysene, and benzo[*b*]fluoranthene) exposure through consumption of coffee, mate, and tea was estimated. The oesophageal cancer risk assessment for both PAH and temperature was conducted using the MOE approach.

**Results:**

Considering differences in the transfer of the PAH from the leaves of mate and tea or from the ground coffee to the infusion, and considering the different preparation methods, exposures may vary considerably. The average individual exposure in μg/kg bw/day arising from consumption of 1 cup (0.2 L) of infusion was highest for mate (2.85E-04 BaP and 7.22E-04 PAH4). The average per capita exposure in μg/kg bw/day was as follows: coffee (4.21E-04 BaP, 4.15E-03 PAH4), mate (4.26E-03 BaP, 2.45E-02 PAH4), and tea (8.03E-04 BaP, 4.98E-03 PAH4). For all individual and population-based exposure scenarios, the average MOE for BaP and PAH4 was > 100,000 independent of beverage type. MOE values in this magnitude are considered as a very low risk. On the contrary, the MOE for the temperature effect was estimated as < 1 for very hot drinking temperatures, corroborating epidemiological observations about a probable oesophageal cancer risk caused by this behaviour.

**Conclusions:**

The temperature effect but not PAH exposure may pose an oesophageal cancer risk. Consumer education on risks associated with consumption of ‘very hot’ beverages and policy measures to threshold serving temperatures should be discussed.

**Electronic supplementary material:**

The online version of this article (10.1186/s12885-018-4060-z) contains supplementary material, which is available to authorized users.

## Background

Consumption of very hot beverages (> 65 °C) is probably associated with an increased risk of oesophageal cancer [1, 2]. The first associations were found for the beverage mate (yerba mate). Mate is a herbal tisane-like beverage widely consumed in some South American countries, where the incidence of oesophageal squamous cell carcinoma (ESSC) is high, and notably higher than in other Latin American countries [3–5]. Currently, mate consumption is also gaining popularity worldwide. The infusion is made from dried leaves of *Ilex paraguariensis* A.St.-Hil. [6]. Many mechanisms for carcinogenicity have been put forward. These include thermal injury, hyperthermia induced endogenous formation of nitrosamines, or impairment of mucosal barrier to entry of potential carcinogenic substances such as polycyclic aromatic hydrocarbons **(**PAH) [7]. Later research on other beverage groups such as *Camelia sinensis* tea and coffee, which are also consumed very hot, resulted in reports of positive associations with increased risk of oesophageal cancer as well [8–11]. Considering that there is little evidence that these beverages contain high amounts of PAH [1], it has become the prevailing hypothesis that the risk might not be inherent to natural compounds contained in mate, or to compounds produced during processing, but rather due to a temperature effect. The first observations of associations between cancer and mate consumption may be explained by the fact that it is typically consumed extremely hot, with temperatures above 70–85 °C in some countries and regions, and in these areas, consumption levels are very high [1, 12]. For this reason, the International Agency for Research on Cancer (IARC) modified its evaluation from “hot mate drinking”, which was classified as probably carcinogenic (group 2A) since 1991 [13], into “very hot beverages at above 65 °C drinking” (group 2A) during its recent monograph (Vol. 116) meeting in 2016 [1]. Nevertheless, an additional role for the PAH contents in mate on oesophageal cancer risk cannot be completely discarded yet.

PAH belong to a large group of over 100 different types of polyarenes that arise from incomplete combustion of organic matter [14–16]. Besides tobacco smoke, diet is the main source of PAH exposure in humans [17]. PAH contamination in food results from processing practices such as smoking, roasting, grilling, or drying during preparation and also from environmental uptake [18]. BaP is the traditional marker for PAH contamination but the European Food Safety Authority (EFSA) in 2008 recommended the use of both BaP and PAH4 (the sum of benzo[*a*]anthracene, chrysene, benzo[*b*]fluoranthene and BaP) as markers for the occurrence and toxicity of PAH in foods [19]. The IARC classifies the compounds in the PAH4 subgroup as follows: BaP is carcinogenic to humans (group 1), while benzo[*a*]anthracene, chrysene and benzo[*b*]fluoranthene are “possibly carcinogenic to humans” (group 2B) [20]. PAH4 is useful for evaluation of products that do not contain BaP. Following this, the EU established a regulation for the maximum levels of PAH4 in some food matrices [21]. However, limits for coffee, mate and tea were not included in the regulation [21]. According to epidemiological findings, the consumption of PAH contaminated food has been linked to the occurrence of malignant tumours of various sites including the oesophagus [3, 22–24].

To disentangle the role of PAH and high temperature, comparative risk assessment of both is needed. The EFSA has developed and recommends an approach known as the margin of exposure **(**MOE), for which doses of substances that have been observed to cause low but measurable harmful responses are compared with relevant substance specific dietary intake estimates in humans.

In this study, we used the MOE methodology to quantitatively assess the risk of PAH from mate, tea, or coffee, in comparison with the risk of high temperatures. We used occurrence data of BaP and PAH4 compounds in coffee, mate and tea (beans and leaves) and infusions to estimate exposure arising from moderate and heavy consumption.

## Methods

### Research on toxicity and occurrence data

Toxicity data on PAH were obtained by a computer-assisted literature search by researchers with qualifications in food science, chemistry, toxicology, epidemiology and cancer risk assessment. Searches were carried out in the following databases: PubMed, Toxnet and ChemIDplus (U.S. National Library of Medicine, Bethesda, MD), Web of Science (Thomson Scientific, Philadelphia, PA), and IPCS/INCHEM (International Programme on Chemical Safety/Chemical Safety Information from Intergovernmental Organizations, WHO, Geneva, Switzerland). Searches on the occurrence of PAH were carried out in August 2016 in the following databases: PubMed (US National Library of Medicine, Bethseda, MD), Web of Science (Thomson Scientific, Philadelphia, PA), Google scholar (Google, NY, USA) and SciELO - Scientific Electronic Library Online (FAPESP - BIREME, São Paulo SP - Brazil) using the key words ‘polycyclic aromatic hydrocarbons’ or ‘PAH AND coffee’ or ‘PAH AND tea’ or ‘PAH AND mate’ or ‘BaP AND coffee’ or ‘BaP AND tea’ or ‘BaP AND mate’ or ‘PAH4 AND coffee’ or ‘PAH4 AND tea’ or ‘PAH4 AND mate’. Additionally, own analytical data of 28 tea, 26 coffee and 9 mate samples were included. The samples were submitted between 2013 and 2016 as official samples in the German Federal State Baden-Württemberg and were analysed using the German reference procedure [25].

Efforts were made to include all available studies; this was accomplished by a hand search of the reference lists of all articles for any relevant studies not included in the databases. The references, including abstracts, were imported into Mendeley (Mendeley Inc., NY, USA) and the relevant articles were manually identified and obtained in full text. We did not identify any article, which was available as abstract only or which we were not able to obtain in full text. No unpublished study was identified.

To provide a meta analysis over all PAH studies, the analytical data of all single studies were combined and descriptive statistics were calculated. No weighting between studies was conducted. The values of non-detectable results were set at the limit of detection to obtain the final distributions. All distributions were separately calculated for the analytical data based on beans/leaves as well as for data based on infusion analyses (i.e. the beverages were analysed after brewing in the final form to be consumed). The infusion data for tea were insufficient to conduct such an analysis, however.

For data on temperature effect, the references cited in IARC monograph Vol. 116 [1] were used and no additional literature searches were conducted.

### Approach for risk assessment

The risk assessment was conducted according to the harmonised approach of the EFSA for the risk assessment of substances that are genotoxic and carcinogenic [26]. The methodology for quantitative risk assessment was based on previous studies for comparative risk assessment of alcohol and drugs [27, 28]. Computation of exposure scenarios considered the amount of leaves/beans used for preparation of infusions in real life [6, 12, 29–31].

The MOE approach was used for the risk assessment [26, 32]. The benchmark dose (BMD), derived from animal cancer data by mathematical modelling within the observed range of experimental data, is recommended as a standardized reference point. To obtain the MOE, the Benchmark Dose Lower Confidence Limit (BMDL) of 10% was selected. The BMDL is an estimate of the lowest dose that is 95% certain to cause no more than a 10% cancer incidence in rodents. In general, BMDLs are used as the statistical lower confidence limits of benchmark doses to derive “safe” exposure levels [33].

The BMD doses for PAH are available in the literature, and the following BMDL values were used as points of departure for the cancer risk assessment: 70 μg/kg bw/day for BaP and 340 μg/kg bw/day for PAH4 based on the most recent EFSA report [19].

For temperature, no BMD modelling results were available in the literature so that own modelling was conducted based on literature studies. No adequate human data for dose-response modelling was identified. Two co-carcinogenicity studies in mice and rats were available. The study in mice reported the incidences of oesophageal lesions (hyperplasia, dysplasia, or oesophageal tumours) induced by *N*-nitrosodiethylamine (NDEA) in the presence of hot water (at 70 °C but not at 60 °C). The mouse study, however, did not provide raw data results suitable for BMD modelling. The study in rats reported data on *N*-nitrosomethylbenzylamine (NMBA)-induced oesophageal tumours, which was significantly increased at 65 °C versus 55 °C and the control group [34].

MOEs were calculated by dividing the reference point, i.e. the BMDL, by the estimated human intakes, with low MOE implying larger risks for humans. A threshold of 10,000 is often used to define public health risks, which considers species differences, human variability and additional uncertainties for substances that are genotoxic and carcinogenic [26].

The BMD(L) values were either taken from the literature search, or additionally BMD and BMDL values were calculated using the US EPA’s BMDS 2.6.0.1 software (available at the US EPA website: http://www.epa.gov/ncea/bmds/index.html). The exposures and MOE were then calculated using the software package @Risk for Excel Version 7.5.0 (Palisade Corporation, Ithaca, NY, USA). Monte Carlo simulations were performed with 10,000 iterations using Latin Hypercube sampling and Mersenne Twister random number generator. The distribution functions and detailed calculation methodology is specified in Additional file 1: Tables S1-S2 online.

### Population-based dietary intake assessment and exposure scenarios

The EFSA harmonized approach has also been used for the dietary intake assessment analysis [35]. The BaP and PAH4 content of coffee, tea and mate were estimated based on data from our literature review. Special exposure scenarios were developed for light, moderate and heavy drinkers. In order to assess individual exposure scenarios, average daily consumption at 1 cup (0.2 L) for light drinking, 2 cups (0.4 L) for moderate drinking, and one and three litres for heavy and very heavy consumers were considered. Figures of per capita consumption for population-based assessments were obtained from Ref. [1]. The PAH exposure due to beverage consumption was then calculated considering these different individual as well as population-based exposure scenarios.

## Results

### Occurrence of BaP and PAH4 in coffee, mate and tea

The occurrence data regarding BaP and PAH4 in coffee, tea and mate from 54 studies in total are summarized in Table 1. The final distributions over all data are provided in Table 2. The average contents of BaP in various matrices were as follows: mate leaves 142.9 μg/kg, tea leaves 8.71 μg/kg, coffee beans 4.12 μg/kg, mate infusion 0.072 μg/L and coffee infusion 0.004 μg/L; while the average content of PAH4 was as follows: mate leaves 872.6 μg/kg, tea leaves 150.53 μg/kg, coffee beans 22.29 μg/kg, mate infusion 0.214 μg/L and coffee infusion 0.041 μg/L (Table 2). Among the matrices of beans and leaves, mate leaves contained the highest amount of BaP and PAH4 while coffee beans had the lowest. The low number of studies on infusions must be noted, which may introduce bias especially as a majority of the infusion studies reported were on tea (*n* = 4, with most samples below the limit of detection making it impossible to make a distribution estimate) compared to only single studies on mate (*n* = 1) and coffee (n = 1).

**Table 1:** Literature data on BaP and PAH4 in coffee, mate and tea from the market and experimental studies

Matrix/Study/(Country)	N	Mean	Median	P90	P95	P99	Maximum	LOD^a^	% > LOD^a^
1. Coffee beans (μg/kg)									
This study (Germany)	26	0.03 /0.88	0 /0	0 /1.85	0 /2.28	0.68 /4.13	0.9 /2.4	0.3	4
[42] (Poland)	28	5.2 /25.2	19.5 /33.8	13.8 /105.6	19.5 / 119.9	31.06 /151.3	9.8 /162.2	0.27	50
[51] (Brazil)	8	0.5 /3.8	0.6/4.3	0.5 /4.5	0.5 / 4.6	0.5 / 4.6	0.51 /4.57	0.07	100
[61] (France)	5	0.028/ 0.066	0 /0.078	0.075 / 0.133	0.085/ 0.147	0.093 /0.159	0.096 / 0.162	0.0008	40
[62] (India)	4	24.6 /79.4	0 /0	68.8 /222.32	83.6 /270.0	95.4/308.1	98.3 /317.6	0.03	25
[63] (Nigeria)	4	25.7 / 31.1	0.03 / 0.73	72.04 /85.69	87.47 / 103.90	99.87 /118.5	102.9 / 122.1	0.03	25
[64] (Romania)	4	0.02 / 0.086	0.02/ 0.088	0.02 /0.1	0.02 /0.1	0.02 /0.1	0.02 /0.1	0.02	0
[19] EFSA	28	2.39 /12	–	–	–	–	–	–	–
[65] (Brazil)	5	0.52 / 1.66	0.43 / 1.49	1.04 /2.79	1.14 /2.91	1.211 /3.00	1.23 /3.03	0.07	80
[50] (Japan)	1	0.53 / 0.68	–	–	–	–	–	–	–
[44] (Brazil)	24	2.10 /−	1.49 /−	4.26 /−	4.47 /−	10.67 /−	12.52 /−	0.03	100
[43] (Portugal)	4	0.32 /6.16	0.29 / 6.23	0.54 / 8.09	0.57 /8.41	0.59 /8.67	0.6 /8.73	–	–
[66] (China)	10	0.12 / 2.52	0.09 / 2.72	0.28 / 4.23	0.32 /4.30	0.35 /4.37	0.36 /4.39	0.031	100
[53] (India)	3	0.33; 2.11	0.28; 2.03	0.42; 2.23	0.44 /2.26	0.45 /2.28	0.46 /2.28	–	–
[35] (France)	2	0 /2.06	–	–	–	–	–	–	–
[67] (USA)	13	7.1 / 186.1	4.9 / 143.4	13.9 / 334.7	15.8 / 236.5	18.0 /395.1	18.5 / 403.2	0.01	92
[49] (Denmark)	11	0.51 / 2.85	0.42 / 2.65	0.88 /4.5	0.94 /4.8	0.99 /5.0	1 /5.1	0.1	100
2. Coffee infusion (μg/L)									
[41] (Brazil)	36	0.004 / 0.041	0 /0.033	0.012 / 0.058	0.014 / 0.076	0.016 /0.095	0.016 /0.099	0.006	11
3. Mate leaves (μg/kg)									
This study (Germany)	9	6.8 / 39	7.6 / 37	9.0 / 57	9.2 / 65	9.4 / 73	9.4 / 74	0.3	100
[38] (not specified)	6	90.2 / 446.7	81.5 /330	150 /770	155 /785	159 /797	160 /800	–	–
[37] (Brazil)	18	11.4 / 116.8	0 /113.2	37.4 / 251.9	44.4 / 257.9	52.6 / 266.6	54.7 / 268.8	0.5	30
[54] (Germany)	2	224.8 / 2117.7	–	–	–	–	–	–	–
[59] (Germany)	8	115.2 / 1075.7	88.0 / 517.3	201.4 / 1019.1	218.9 / 1047.4	233.0 / 1070.0	236.5 / 1075.7	0.01	100
[68] (Germany)	3	200 /800	240 / 940	272 / 1308	276 / 1354	279.2 / 1390.8	280 / 1400	–	–
[69] (China)	1	542.3 / 2736.8	–	–	–	–	–	–	–
[39] (Brazil)	12	31.7 / 133.1	19.8 / 111.5	61.2 / 254.0	79.0 / 283.9	95.2 / 301.0	99.3 / 305.3	–	–
[36] (Brazil)	8	50 / 355.7	40.1 / 319.2	52.7 / 368.3	53.0 / 383	53.2 / 394.8	53.3 / 397.7	–	–
4. Mate infusion (μg/L)									
[12] (Brazil)	20	0.13 / 0.39	0.04 / 0.24	0.24 / 0.93	0.42 / 1.08	1.05 / 1.61	1.22 / 1.74	0.0012	100
[46] (Brazil)	11	0.014 / 0.038	0.012 / 0.033	0.014 / 0.059	0.014 / 0.087	0.014 /0.110	0.022; 0.116	0.001–0.009	–
5. Tea leaves (μg/kg)									
This study (Germany)	28	10.5 / 30.1	4.9 / 16.4	15.8 / 41.1	19.2 / 51.4	96.9 / 249.3	125 / 321.4	0.3	100
[19] EFSA	30	8.38 / 42.67	–	–	–	–	–	–	–
[70] (India)	5	6.1 /6.1	0 /0	15.6 / 15.6	16.1 /16.1	16.5 /16.5	16.6 /16.6	–	–
[50] (Japan)	8	14.8 /49.2	5.6 /25.6	33.9 / 107.6	53.3 /156.4	69.3 /195.4	73.2 /205.1	0.53	88
[71] (Germany)	18	3.59 / 13.59	–	–	–	–	–	0.26	–
[72] (China)	1	18.2 /32.8	–	–	–	–	–	0.001	100
[69] (China)	6	11.6 / 118.1	0 /18.7	34.7 / 332.8	47.8 /345.9	58.4 /356.4	61 /359	–	–
[73] (China)	4	10.1 / 127.2	10.2 /126.0	12.0 / 172.2	12.3 /173.4	12.5 /174.5	12.6 /174.7	–	–
[54] (Germany)	5	3.3 /57.7	3.14 /73.7	5.7 /95.0	5.8 /99.4	5.9 /102.8	5.9 /103.7	–	–
[59] (Germany)	25	8.0 /59.8	5.3 /38.6	17.0 / 147.9	18.7 /153.2	29.34 /224.6	32.6 / 246.8	0.01	100
[36] (Brazil)	1	4.24 /−	- /−	- /−	- /−	- /−	- /−	–	–
[74] (Iran)	8	0 /97.9	0 /89.0	0 /216.5	0 / 279.1	0 /329.2	0 /341.7	–	–
[75] (Argentina)	54	9.9 /51.2	7.7 /39	14.9 /74.9	18.2 /83.5	20.8 /90.3	21.5 /92	–	–
[55] (France)	15	4.2 /79.7	2.8 /54.4	8.2 /178.7	13.0 /191.2	20.1 /212.6	21.9 /218	0.6	80
[42] (Poland)	22	0 /17.7	0 /15.5	0 /32.3	0 /43.1	0 /50.9	0 /20.9	0.3	0
[76] (Czech Republic)	36	14.3 /82.7	14.3 /82.7	20.3 / 109.3	21.0 /112.6	21.6 /115.2	21.8 /115.9	–	–
[77] (China)	12	18.8 / -	10.3 / -	38.2 / -	52.1 / -	64.6 / -	67.7 / -	0.28	100
[48] (USA)	28	5.9 /116.3	3 /102.5	12.6 / 242.7	14.0 /362.5	25.0 /384.7	29.0 /426.0	0.01	100
[49] (Denmark)	10	12.8 /57.5	–	–	–	–	–	–	–
[63] (Nigeria)	9	18.2 /35.4	0 /27.2	48.9 / 72.0	93.0 /104.5	128.3 /130.6	137.1 / 137.1	0.03	11
[53] (India)	5	0 /1784.5	–	–	–	–	–	–	–
[68] (Germany)^b^	56	38.0/166.2	6.2/35.5	67.0/325	285/1050	388.5/ 1645	460/ 1700	0.05	100
6. Tea infusion (μg/L)									
[78] (Spain)	7	0.01 /0.04	0.007/ 0021	0.018/ 0.0823	0.021/0.094	0.023/ 0.103	0.024/ 0.105	0.024	14.3
[68] (Germany)	14	< 0.005 / < 0.005	–	–	–	–	–	0.005	0
[55] (France)	10	0.1/1.1	0.1/0.8	0.1/1.7	0.1;2.2	0.1/2.6	0.1/ 2.7	0.6	0
[53] (India)	5	0/4.5	0/4.0	0/6.0	0/6.3	0/6.5	0/6.6	0.22	0

Values before and after / are for BaP and PAH4, respectively; values marked as (−) not calculable because raw data is not available; values reported in μg/kg for leaves/beans and μg/L for infusions;

^a^LOD values are for BaP

^b^not included in calculation due to data assignment error

**Table 2:** Meta-analysis on BaP and PAH4 occurrence in coffee, mate and tea

	BaP (PAH4)						
Matrix	Data source(s)	N	Mean	Median	P90	P95	P99
Coffee beans	[This study, 18,30–32,36–38,40,52–58]	180	4.12 /22.29	1.65 /12.34	10.39 / 48.55	12.64 /55.38	15.34 /62.83
Coffee infusion	[41]	36	0.004 /0.041	0 /0.033	0.012 / 0.058	0.014 /0.076	0.016 /0.095
Mate leaves	[This study, 4,5,50,59,51,60–62]	59	142.9 /872.6	78.2 /388.5	129.1 / 661.9	137.7 /685.1	219.3 /703.2
Mate infusion	[12, 46]	31	0.072 /0.214	0.026 /0.137	0.127 / 0.495	0.217 /0.584	0.532 /0.860
Tea leaves	[This study, 4,18,30,35–37,40,47,50,54,51,61,63–70]	330	8.71 /150.53	7.84 /50.66	18.52 / 131.33	25.63 / 148.52	37.95 /272.03
Tea infusion^a^	[53, 55, 68, 78]	36	–	–	–	–	–

Values before and after / are for BaP and PAH4, respectively; values marked as (−) not calculable because raw data is not available; values reported in μg/kg for leaves/beans and μg/L for infusions. ^a^No distribution calculated for tea infusion because most values were below LOD

### Exposure assessment

The exposure for different individual and population-based scenarios was separately calculated for the analytical data based on beans/leaves considering the extraction percentage (Table 3) and the data based on direct analyses of infusions. The exposure scenarios are summarized in Table 4. The full distributions are provided in Additional file 1: Table S3.

**Table 3:** Extraction of PAH to beverage

Matrix	% extraction^a^			PAH	Reference
	Minimum	Maximum	Mean		
Coffee	0.8	1	0.9	BaP	[79]
Tea	3.6	5.5		BaP	[80]
	4.5	6.8		PAH4	
Tea	0	0.52		PAH	[68]
Coffee	0.6	26	5	BaP	[81]
Tea	0	2.3	0.86	PAH4	[49]
Coffee (Dark roasted)	0	14	9	PAH4	[34]
Coffee (Medium roasted)	5	9	7	PAH4	
Mate	6	37		PAH	[36]
			50	BaP	
Mate	2	6			[69]
Black tea	3	50	7.7	PAH	[24]
Tea	1.1	3.1		BaP	[50]
Tea	82	123		PAH	[55]

^a^Extraction in this context means the transfer of PAH from the solid material (beans or tea leaves) into the beverages during preparation. The data were obtained from experimental studies in the literature

**Table 4:** Exposure and margin of exposure of BaP and PAH4 in different exposure scenarios

Matrix	Light drinking (1 cup - 0.2 L)		Moderate drinking (2 cups - 0.4 L)		Heavy drinking (1 L)		Very heavy drinking (3 L)		Daily per capita	
	BaP	PAH4	BaP	PAH4	BaP	PAH4	BaP	PAH4	BaP	PAH4
	Mean/P95	Mean/P95	Mean/P95	Mean/P95	Mean/P95	Mean/P95	Mean/P95	Mean/P95	Mean/P95	Mean/P95
A. Exposure (µg/kg bw/day)										
1. Coffee										
Beans	1.88E-04/5.95E-04	1.85E-03/5.77E-03	3.77E-04/1.19E-03	3.70E-03/1.15E-02	9.42E-04/2.97E-03	9.26E-03/2.89E-02	2.83E-03/8.92E-03	2.78E-02/8.66E-02	4.21E-04/1.32E-03	4.15E-03/1.28E-02
Infusion	1.71E-04/5.21E-04	1.00E-03/3.10E-03	3.41E-04/1.04E-03	2.01E-03/6.20E-03	8.54E-04/2.61E-03	5.02E-03/1.55E-02	2.56E-03/7.82E-03	1.51E-02/4.65E-02		
2. Mate										
Leaves	1.94E-03/6.10E-03	1.11E-02/3.47E-02	3.87E-03/1.22E-02	2.22E-02/6.94E-02	9.68E-03/3.05E-02	5.54E-02/1.73E-01	2.90E-02/9.15E-02	1.66E-01/5.20E-01	4.26E-03/1.44E-02	2.45E-02/8.10E-02
Infusion	2.85E-04/8.62E-04	7.22E-04/2.18E-03	5.70E-04/1.72E-03	1.44E-03/4.35E-03	1.42E-03/4.31E-03	3.61E-03/1.09E-02	4.27E-03/1.29E-02	1.08E-02/3.26E-02		
3. Tea										
Leaves	1.65E-04/5.12E-04	1.02E-03/3.26E-03	3.30E-04/1.02E-03	2.04E-03/6.52E-03	8.26E-04/2.56E-03	5.11E-03/1.63E-02	2.48E-03/7.68E-03	1.53E-02/4.89E-02	8.03E-04/2.78E-03	4.98E-03/1.68E-02
Infusion	–	–	–	–	–	–	–	–		
B. Margin of exposure										
1. Coffee										
Beans	4.48E+06/7.98E+06	2.18E+06/4.07E+06	2.24E+06/3.99E+06	1.09E+06/2.04E+06	8.97E+05/1.60E+06	4.35E+05/8.14E+05	2.99E+05/5.32E+05	1.45E+05/2.71E+05	1.97E+06/3.48E+06	9.70E+05/1.72E+06
Infusion	4.61E+06/8.21E+06	5.01E+06/6.72E+06	2.30E+06/4.11E+06	2.51E+06/3.36E+06	9.21E+05/1.64E+06	1.00E+06/1.34E+06	3.07E+05/5.48E+05	3.34E+05/4.48E+05		
2. Mate										
Leaves	4.97E+05/7.54E+05	1.13E+06/6.83E+05	2.49E+05/3.77E+05	5.67E+05/3.42E+05	9.94E+04/1.51E+05	2.27E+05/1.37E+05	3.31E+04/5.03E+04	7.56E+04/4.56E+04	4.91E+05/3.74E+05	2.31E+05/4.32E+05
Infusion	2.45E+06/4.97E+06	6.85E+06/9.34E+06	1.22E+06/2.48E+06	3.43E+06/4.67E+06	4.89E+05/9.93E+05	1.37E+06/1.87E+06	1.63E+05/3.31E+05	4.57E+05/6.23E+05		
3. Tea										
Leaves	4.50E+06/8.96E+06	3.35E+06/7.15E+06	2.25E+06/4.48E+06	1.68E+06/3.58E+06	9.00E+05/1.79E+06	6.71E+05/1.43E+06	3.00E+05/5.97E+05	2.24E+05/4.77E+05	1.39E+06/2.47E+06	1.13E+06/1.83E+06
Infusion	–	–	–	–	–	–	–	–		

Based on results for consumption of 1 cup (0.2 L) using the data for beans and leaves analyses, mate had the highest BaP exposures at 1.94E-03 μg/kg bw/day followed by coffee at 1.88E-04 μg/kg bw/day. Similarly, PAH4 exposure was highest for mate, 1.11E-02 μg/kg bw/day, and lowest for tea, 1.02E-03 μg/kg bw/day. For infusion analyses, the BaP exposure was highest for mate, 2.85E-04 μg/kg bw/day, and lowest for coffee while PAH4 exposure was lowest for mate, 7.22E-04 μg/kg bw/day as shown in Table 4. The per capita consumption data are reported as amount of beans/leaves only, so that for this approach only the analytical data from beans/leaves were used. The per capita exposures were as follows: coffee (4.21E-04 μg/kg bw/day BaP and 4.15E-03 μg/kg bw/day PAH4), mate (4.26E-03 μg/kg bw/day BaP and 2.45E-02 μg/kg bw/day PAH4), tea (8.03E-04 μg/kg bw/day BaP and 4.98E-03 μg/kg bw/day PAH4) as shown in Table 4.

### Benchmark dose (BMD) modelling for temperature

The Additional file 2: data appendix provided as supplementary material shows the raw results of benchmark dose-response modelling of a study in rats. The results are summarized in Table 5. The BMD for NMBA-induced oesophageal tumours was 64 °C and the BMDL was 56 °C (Table 5).

**Table 5:** Toxicological thresholds of PAH and temperature

Agent	Species	Effect	BMD	BMDL	Reference
BaP	Mice, 2 year study	Tumours of the alimentary tract	0.13–0.14 mg/kg bw/day	70 μg/kg bw/day	EFSA [19]
PAH4	Mice, 2 year study	Tumours of the alimentary tract	0.60–0.61 mg/kg bw/day	340 μg/kg bw/day	EFSA [19]
Temperature	Rats, 20 week study	NMBA-induced oesophageal tumours (mean number of tumours)	64 °C	56 °C	Own modelling^a^ based on data from Li et al. 2003 [34]
Temperature	Rats, 20 week study	NMBA-induced oesophageal tumours (mean volume of tumours)	(63 °C)^b^	(26 °C)^b^	Own modelling^a^ based on data from Li et al. 2003 [34]

^a^See data Additional file: appendix provided as supplementary material for raw results of benchmark dose-response modelling

^b^Non-significant difference from control group according to original reference (Li et al. 2003) [34]

### Cancer risk assessment using the margin of exposure (MOE) approach

The MOE values for the different scenarios are summarized in Table 4. The full distributions are provided in Additional file 1: Table S3.

Based on results for consumption of 1 cup (0.2 L) in the matrices of beans and leaves, tea had the average highest MOE from BaP at 4.50E+06 followed by coffee at 4.48E+06 while MOE from PAH4 was highest for tea, 3.35E+06 and lowest for mate, 1.13E+06. For infusions, the BaP MOE was highest for coffee 4.61E+06 and lowest for mate 2.45E+06 while MOE from PAH4 was highest for mate, 6.85E+06 as shown in Table 4.

The per capita MOE was in the order of 1.97E+06, 1.39E+06, 4.91E+05 for coffee, tea and mate, respectively, and at the 95th percentile the order of MOE does not change (Table 4).

**Fig. 1:**
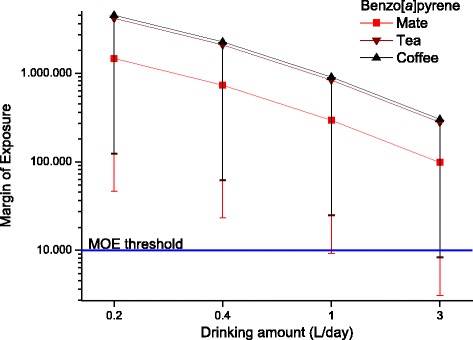
Margin of Exposure for benzo[*a*]pyrene calculated for different exposure scenarios for drinking mate, tea and coffee (calculated as average for data based on leaves/beans and infusion analyses, mean with 5th percentile as error bar are shown)

**Fig. 2:**
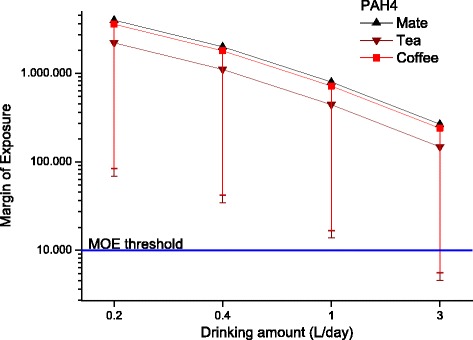
Margin of Exposure for PAH4 calculated for different exposure scenarios for drinking mate, tea and coffee (calculated as average for data based on leaves/beans and infusion analyses, mean with 5th percentile as error bar are shown)

**Fig. 3:**
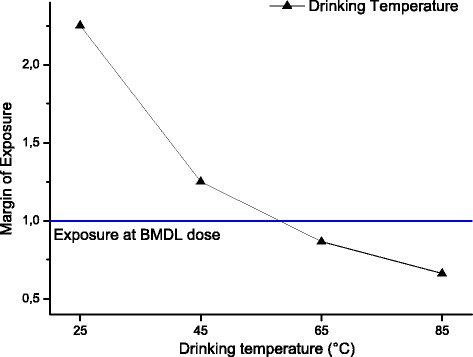
Estimation of Margin of Exposure for drinking temperature between 25 and 85 °C (calculated as point estimate based on BMDL from a co-carcinogenicity study in rats. No error bars available due to lack of data. No distribution available for BMDL because only one single study for modelling)

The margins of exposure for BaP (Fig. 1) and PAH4 (Fig. 2) for the different individual drinking scenarios are compared to the MOE threshold of 10,000. Only the extreme consumption of 3 L per day (meaning that the complete daily liquid requirement is fulfilled with these hot beverages) in worst-case scenarios (95th percentile contamination) would lead to MOEs below this threshold for all three beverages. Due to the high variance in the distributions, no significant beverage-specific influence was detected. Figure 3 shows the margin of exposure estimated for temperature. For 25 °C and 85 °C, the MOE would be 2.3 and 0.7, respectively. Hence, the MOE is below 1 at very hot temperatures.

## Discussion

The competing role of PAH and hot temperatures in hot beverages of widespread consumption has been hard to disentangle. This study reports a comprehensive worldwide systematic overview of the cancer risk assessment for PAH in comparison with very hot (> 65 °C) coffee, tea and mate. To our knowledge, this is the first study to quantitatively estimate cancer risk of PAH in mate compared to coffee and tea, and also to provide a comparison with the risk of the temperature effect using the margin of exposure methodology.

Mate is traditionally consumed either as hot mate or cold mate. The hot mate infusion consumed in South America is made by placing 20–50 g of mate in a vessel where very hot water (70–85 °C) is slowly poured over the material and after each pouring, the water is sucked through a special drinking straw fitted with a filter on the end immersed in the mate infusion [6, 12, 31]. Cold mate may be consumed in the same way or in a glass like a regular drink, at 4–8 °C [12]. PAH in mate are produced by fire or high temperature exposure during traditional drying using direct fire and/or during roasting of the mate leaves [36–40]. The commercial processing of mate may involve two successive drying stages namely an initial rapid drying process at 400–750 °C by use of direct flames followed by final drying at 90–350 °C in rotating cylinders heated by burning wood before grinding [37].

PAH in the widely consumed beverage tea (*Camellia sinensis)* may result from air pollution in tea farms and during processing which may involve drying over burning wood, coal or oil [24]. Coffee is also another non-alcoholic beverage consumed worldwide, and another possible source of PAH that can form through roasting, which is a crucial step in the processing of coffee for the development of aroma, flavour and colour [35, 41–43]. In green coffee, however, both absence and presence of PAH have been reported [43–45] and the eventual presence has been associated with air pollution rather than the high temperatures used during drying of the seeds.

Our finding of MOE values of more than 100,000 for PAH and less than 1 for very hot temperatures provides quantitative confirmation that “very hot” drinking temperatures are probably causally related to oesophageal cancer whereas low temperature mate is not. The very high MOE for PAH, which suggests that the human dosage is considerably below the lowest effective dose in animals, also makes the assumption implausible that PAH and temperature may act synergistically. Moreover, the very similar PAH exposure levels from mate and tea consumption offer indirect further support for IARC’s evaluation. In countries, such as the UK, where tea is consumed typically at lower temperature than the one of mate consumption in South America, increased rates of oesophageal cancer have not been observed. Also, it might be a matter of temperature dosage, mate drinkers might be drinking many more drinks per day than tea drinkers that possibly drink 1–2 cups per ‘session’, whereas a hot mate drinker can easily drink 1 l worth of water in a ‘session’, thus exposing the oesophagus many more times to very hot temperatures. Our assessment excludes assumptions of a beverage-specific effect, and confirms the limited epidemiological evidence that suggested a risk for very hot beverages independent of type. The mechanism of carcinogenicity of very hot beverages deserves further studies.

A limitation of our study is the fact that for some jurisdictions only a sparsity of data was available for PAH. Apart from Germany where data for all the beverage categories was available, most studies in other countries focused on only one type of beverage probably due to high prevalence of consumption in those countries. There were only extremely limited studies on PAH in infusions available, for mate and coffee only one single study. Due to the lack of data, even such single studies were included in our meta-analysis, specifically because we judge such direct beverage analyses more meaningful than bean/leaves analyses. However, we note that the same limitations generally apply to the assessments of the international agencies EFSA and Food and Agriculture Organization/WHO Expert Committee on Food Additives (JECFA), whose approaches we specifically followed.

In some samples, the occurrence of BaP was reported to be zero or below the limit of detection and this necessitated the use of the LOD value in order to calculate descriptive statistics. The sensitivity of the analytical methods used could explain the different LOD values reported. Analysis of PAH in foods such as coffee, mate and tea is complex due to the many compounds present in such food matrices, requiring sample preparative procedures such as liquid-liquid extraction (LLE), solid phase extraction (SPE), liquid phase microextraction (LPME), membrane assisted solvent microextraction, solid phase microextraction (SPME) and stir bar sorptive extraction (SBSE) [46]. The extraction processes not only make the analysis expensive but may also compromise on recovery of PAH [14]. Coupling the preparation step with analytical equipment is feasible such as SPME-HPLC or head space SPME-HPLC [47]. Separation and quantification of the PAH in infusions is by liquid chromatography with fluorescent detection (LC-FLD) [12, 41, 48] or by GC-MS [49], while in ground coffee LC-FLD [44, 50], isotope dilution GC-MS [51], GC-MS/MS [52], or LC-UV [53], and in tea leaves, LC-UV [53], or GC-MS [49] have been utilized.

The substitution of “non detectable” data with LOD is a conservative approach taken and therefore the risk would have been overestimated (in relation to the approach of setting such values as 0). Some studies had limited sample sizes as low as 1 and this could skew meta-analysis results. Nevertheless, we think our distributions are robust. For example, no different judgments would arise with the calculation method of setting non detectable values as 0 (data not shown).

Most studies report the occurrence of PAH in mate and tea leaves and coffee beans (*n* = 48; 89%). However, since the raw leaves and coffee are usually not directly ingested, estimation of PAH concentrations in the beverages is necessary for exposure estimation. The differences in the manner of processing of the leaves for mate and tea and coffee beans and preparation of the beverages influences the levels of PAH in the beverages. The contribution of individual PAH to the total PAH concentration is dependent on its solubility. While the physicochemical data suggest that BaP and PAH are poorly soluble in water, the literature reports very variable extraction percentages from zero to above 50%. Furthermore, it must be considered that hot mate is typically consumed by pouring hot water on the same leaves repeated times, and very often replacing some of the leaves by fresh ones to keep the mate ‘strong’, therefore, the actual amount extracted from the leaves might be higher for mate than for the other beverages that are extracted only once. The actual preparation processes also influence the levels of PAH found in the drink, for example, low levels were found in cold mate compared to hot mate infusions [12]. Literature reports extraction of BaP and PAH4 in the range 0.6–50% and 0–123% respectively (Table 3). The high transfer in tea reported has been linked to its high contents of essential oils, which may act as co-solvents that alter the physical and chemical properties of the water used for tea making and increase the affinity of PAH for the aqueous infusion [54, 55].

An investigation by Lin et al. [24] to identify the factors affecting the transfer of PAH singled out tea variety, tea/water ratio, and brewing time to affect the levels. The transfer average of all studies was 27% (which is very similar to the value of 26% used by EFSA for coffee [19]), but due to the probabilistic calculation method, the whole range of literature data was taken into account in our case. Another problem is the variety of brewing methods and amounts of tea leaves or coffee beans used per L of water. This may considerably vary depending on brewing method and individual preferences (e.g. see overview of brewing methods in [1]). For this reason, we have defined reasonable minimum and maximum values and used “no-knowledge” distributions in the probabilistic calculations to randomly consider the whole range of brewing methods. The appropriateness of this approach has been indirectly validated because the resulting exposure data are in reasonable agreement between both calculations methods (based on direct infusion analyses and recalculated from beans/leaves), so that we decided to average the values from both methods for the final data presentation (Figs. 1 and 2).

The higher average content of PAH and consequently the exposures due to mate and tea could be due to the similarities in processing of leaves that involve roasting over wood and that the leaves have larger surface areas that accumulate PAH from air [56]. The age of the raw leaves collected from the farms also influences the level of PAH with the older cultivated mate having higher PAH as a result of longer exposure to environmental contamination [9].

Low amounts of low toxicity PAH have been identified in green coffee, mostly due to exposure to air pollution during sun drying of the beans. The roasting methods can also affect PAH formation in coffee. Drum roasters over direct fire enables uneven and higher temperatures in some parts of the beans while in fluidized bed roasters, frequently used currently, roasting temperatures do not exceed 240–260 °C, never reaching the temperature used for scorching mate. In general, the amount of PAH in coffee beans tend to increase in very dark roasts, only, when the beans are almost carbonized [43]. Due to their low solubility, PAH concentrations in the beverage should be low. Paper filter should retain lipophilic compounds as opposed to extraction methods that do not use such filters such as espresso, Italian (Mocha), Turkish and Scandinavian boiled coffee [57]. Higher amounts of PAH, however, have been identified in torrefacto coffee (roasted with sugar) [58]. There was lack of data for PAH4 in some studies besides BaP; and in one study, occurrence of chrysene in tea leaves was reported as a sum of chrysene and triphenylene due to lack of resolution between the two PAH [59]. However, this did not lead to higher values of PAH compared to other studies. Nevertheless, it could have contributed to risk overestimation in tea.

Compared to the large number of studies on PAH in hot beverages, and also to the toxicity data on PAH available, there is a sparsity of data on the temperature effect.

The weak data basis for thresholds for temperature are because temperature effect studies rely on self-reporting and there is subjectivity in the notion of different temperatures [5]. The human epidemiological data provide evidence of a probable association between very hot beverages and oesophageal cancer, suggesting ~ 2-fold increase with various hot drinks [1, 2]. Importantly, a large pooled analysis was conducted that reported estimates of association between mate drinking and oesophageal cancer stratifying by temperature and showed little evidence of association when mate was consumed warm, regardless of the amount consumed, thus supporting a role of very hot temperatures and not mate per se [3]*.*

Only two animal experiments were judged by IARC as adequate for evaluation, but they are only co-carcinogenicity studies. However, the animal experiments provide excellent corroborating evidence into a threshold, and the BMD of the animal experiment is in excellent agreement with the judgment of the IARC experts, that “very hot beverages” leading to a concern for oesophageal cancer, may start above 65 °C. However, there exists the problem of risk extrapolation from animal to human data especially in this case. The animals in the Li et al. [34] study received very hot water with an amount of 1 mL/kg for 5 times per week. Extrapolated to a human with a bodyweight of 60 kg this would amount to 60 mL of very hot beverage, which is not an unreasonable assumption, even less than one cup size. For this reason, we think that the temperature threshold of the animal study may be directly transferrable to humans. The normal practise in regulatory toxicology would be to add safety factors of 10 for both inter-species extrapolation and for intra-species differences to suggest acceptable daily intakes (ADI). In this case, we do not believe that it would make any sense to add safety factors for example to the BMD of 64 °C to derive such an ADI, because the ADI would clearly lie in a temperature zone (room temperature and below) that is perceived as being completely without risk. Till better studies become available, we think it is currently sensible to use the threshold value of IARC of 65 °C as “acceptable temperature”, which is also in excellent agreement to the BMD of 64 °C calculated in this study.

## Conclusion

PAH are ubiquitous contaminants present in air, soil, water, foods; therefore, their role in carcinogenesis cannot be overlooked. The PAH exposure arising from consumption of coffee, mate and tea to cumulative exposure is rather small, according to EFSA, less than 10% of the combined exposure from all food groups. Nevertheless, contamination regulations usually demand that PAH contamination should be as low as technologically achievable; therefore, any amount of contamination in beverages should be avoided. Considering the limitations of the toxicological data but in light of the epidemiological evidence, the authors believe that risk management should be prioritized regarding the temperature problem rather than the PAH problem. Potential measures may include consumer education to drink at lower temperatures. However, similarly to alcohol consumption, changing behaviours about beverage temperatures might be difficult due to cultural traditions and preferences, since consumers may be more willing to tolerate higher risks for intentional behaviours (such as high temperature drinking) than for unintentional risks (such as PAH contamination) [60]. Considering the increasing use of commercial hot beverage preparation machines, policy measures regarding serving temperatures (e.g. maximum temperatures for commercial hot beverage preparation machines) could also be effective.

## Additional files

Additional file 1 Supplementary Tables S1-S3. Supplementary Table S1. Distribution functions as input for probabilistic analysis; Supplementary Table S2. Detailed calculation methodology for probabilistic risk assessment of PAH due to beverage consumption; Supplementary Table S3. Raw results of probabilistic estimation of BaP and PAH4 exposure and margin of exposure (MOE) using 10,000 iterations. (DOC 250 kb)

Additional file 2 Data appendix with raw result for benchmark dose-response modelling. Supplementary Data S1. BMD modelling for mean number of tumours per rat from Li et al. (2003); Supplementary Data S2. BMD modelling for mean volume of tumours (mm^3^) from Li et al. (2003). (DOC 81 kb)

## Abbreviations

ADI: Acceptable daily intake
BaP: Benzo[*a*]pyrene
BMD: Benchmark dose for a benchmark response of 10%
BMDL/BMDL10: Benchmark Dose Lower Confidence Limit for a benchmark response of 10% (The BMDL is an estimate of the lowest dose that is 95% certain to cause no more than a 10% effect)
Bw: Bodyweight
Chr: Chrysene
EFSA: European Food and Safety Authority
ESCC: Esophageal squamous cell carcinoma
EU: European Union
FLD: Fluorescence detection
GC-MS: Gas chromatography-mass spectrometry
GC-MS/MS: Gas chromatography-tandem mass spectrometry
IARC: International Agency for Research on Cancer
IPCS/INCHEM: International Programme on Chemical Safety/Chemical Safety Information
JECFA: Joint FAO/WHO Expert Committee on Food Additives
LC: Liquid chromatography
LLE: Liquid-liquid extraction
LPME: Liquid phase microextraction
MOE: Margin of exposure
PAH: Polycyclic aromatic hydrocarbons
PAH4: sum of benzo[*a*]pyrene, chrysene, benzo[*b*]fluroanthracene and benzo[*a*]anthracene
SBSE: Stir bar sorptive extraction
SPE: Solid phase extraction
SPME: Solid phase microextraction
TEF: Toxic Equivalent Factors
US EPA: Unites States Environmental Protection Agency
UV: Ultraviolet detector

## References

[1] IARC. IARC Monographs on the Evaluation of Carcinogenic risks to Humans, Vol. 116, Drinking coffee, maté, and very hot beverages. Lyon, France; in press.

[2] Loomis D, Guyton KZ, Grosse Y, Lauby-Secretan B, El Ghissassi F, Bouvard V (2016). Carcinogenicity of drinking coffee, mate, and very hot beverages. Lancet Oncol.

[3] Lubin JH, De Stefani E, Abnet CC, Acosta G, Boffetta P, Victora C (2014). Mate drinking and esophageal squamous cell carcinoma in South America: pooled results from two large multicenter case-control studies. Cancer Epidemiol Biomark Prev.

[4] Fagundes RB, Abnet CC, Strickland PT, Kamangar F, Roth MJ, Taylor PR (2006). Higher urine 1-hydroxy pyrene glucuronide (1-OHPG) is associated with tobacco smoke exposure and drinking maté in healthy subjects from Rio Grande do Sul, Brazil. BMC Cancer.

[5] Szymańska K, Matos E, Hung RJ, Wünsch-Filho V, Eluf-Neto J, Menezes A (2010). Drinking of maté and the risk of cancers of the upper aerodigestive tract in Latin America: a case-control study. Cancer Causes Control.

[6] Heck CI, De Mejia EG (2007). Yerba mate tea (Ilex Paraguariensis): a comprehensive review on chemistry, health implications, and technological considerations. J Food Sci.

[7] Chen Y, Tong Y, Yang C, Gan Y, Sun H, Bi H (2015). Consumption of hot beverages and foods and the risk of esophageal cancer: a meta-analysis of observational studies. BMC Cancer.

[8] Nubia M, Victoria CG, Crespi M, Saul C, Braga NM, Correa P (1987). Hot mate drinking and precancerous lesions of the oesophagus: an endoscopic survey in southern Brazil. Int J Cancer.

[9] Rolón PA, Castellsagué X, Benz M, Hot MN (1995). Cold mate drinking and esophageal cancer in Paraguay. Cancer Epidemiol Biomark Prev.

[10] Castellsagué X, Muñoz N, De Stefani E, Victora CG, Castelletto R, Rolón PA (2000). Influence of mate drinking, hot beverages and diet on esophageal cancer risk in South America. Int J Cancer.

[11] Victora CG, Munoz N, Day NE, Barcelos LB, Peccin DA, Braga NM (1987). Hot beverages and esophageal cancer in southern Brazil: a case- control study. Int J Cancer.

[12] Thea AE, Ferreira D, Brumovsky LA, Polycyclic SME (2016). Aromatic hydrocarbons (PAHs) in yerba mate ( Ilex Paraguariensis St. Hil ) traditional infusions (mate and terere). Food Control.

[13] IARC. IARC Monographs on the evaluation of Carcinogenic risks to Humans Vol. 51, coffee, tea, maté, methylxanthines and methylglyoxal. Lyon, France. 1991. Available online: http://monographs.iarc.fr/ENG/Monographs/vol51/mono51.pdf PMC7681554 1674554

[14] Wenzl T, Simon R, Anklam E, Kleiner J (2006). Analytical methods for polycyclic aromatic hydrocarbons (PAHs) in food and the environment needed for new food legislation in the European Union. TrAC - Trends Anal Chem.

[15] Howard JW, Fazio T (1969). Review of polycyclic aromatic hydrocarbons in foods. J Agric Food Chem.

[16] Mumtaz MM, George JD, Gold KW, Cibulas WDC (1996). ATSDR evaluation of health effects of chemicals. IV. Polycyclic aromatic hydrocarbons (PAHs): understanding a complex problem. Toxicol Ind Health.

[17] Xia Z, Duan X, Qiu W, Liu D, Wang B, Tao S (2010). Health risk assessment on dietary exposure to polycyclic aromatic hydrocarbons (PAHs) in Taiyuan, China. Sci Total Environ.

[18] Bansal V, Review KK (2015). Of PAH contamination in food products and their health hazards. Environ Int.

[19] Authority EFS. Polycyclic aromatic hydrocarbons in food. EFSA J. 2008;724. https://doi.org/10.2903/j.efsa.2008.724.

[20] IARC. List of classifications, Volumes 1–119. http://monographs.iarc.fr/ENG/Classification/latest_classif.php.

[21] European Commission (2011). Commission regulation (EU) no 835/2011 of 19 august 2011 amending regulation (EC) no 1881/2006 as regards maximum levels for polycyclic aromatic hydrocarbons in foodstuffs. Off J Eur Union.

[22] Denissenko MF, Pao A, Tang M, Pfeifer GP (1996). Preferential formation of benzo[a]pyrene adducts at lung cancer mutational hotspots in P53. Science.

[23] Shen J, Terry MB, Gammon MD, Gaudet MM, Teitelbaum SL, Eng SM (2006). IGHMBP2 Thr671Ala polymorphism might be a modifier for the effects of cigarette smoking and PAH - DNA adducts to breast cancer risk. Breast Cancer Research Treat.

[24] Olsson AC, Fevotte J, Fletcher T, Cassidy A, Mannetje A, Zaridze D (2010). Occupational exposure to polycyclic aromatic hydrocarbons and lung cancer risk : a multicenter study in Europe. Occup Env Med.

[25] Anon. Amtliche Sammlung von Untersuchungsverfahren. BVL L 07.00–40. Untersuchung von Lebensmitteln - Bestimmung von Benzo(a)pyren in geräucherten und mit Raucharomen hergestellten Fleischerzeugnissen. Beuth-Verlag, Berlin, Germany. 2004.

[26] EFSA. Opinion Of the scientific committee on a request from EFSA related to a harmonised approach for risk assessment of substances which are both genotoxic and carcinogenic. EFSA J. 2005;282:1–31. https://doi.org/10.2903/j.efsa.2005.282.

[27] Lachenmeier DW, Rehm J (2015). Comparative risk assessment of alcohol, tobacco, cannabis and other illicit drugs using the margin of exposure approach. Sci Rep.

[28] Pflaum T, Hausler T, Baumung C, Ackermann S, Kuballa T, Rehm J (2016). Carcinogenic compounds in alcoholic beverages: an update. Arch Toxicol.

[29] ISO 3103:1980. Tea - Preparation of liquor for use in sensory tests. Geneva: International Organization for Standardization. https://www.iso.org/standard/8250.html.

[30] ISO 6668:2008. Green coffee - Preparation of samples for use in sensory analysis. Geneva: International Organization for Standardization. https://www.iso.org/standard/44609.html.

[31] da Silveira TFF, Meinhart AD, Ballus CA, Godoy HT (2014). The effect of the duration of infusion, temperature, and water volume on the rutin content in the preparation of mate tea beverages: an optimization study. Food Res Int.

[32] EPA (1995). The use of the benchmark dose approach in health risk assessment.

[33] Kodell RL (2009). Replace the NOAEL and LOAEL with the BMDL01 and BMDL10. Environ Ecol Stat.

[34] Li ZG, Shimada Y, Sato F, Maeda M, Itami A, Kaganoi J, et al. Promotion effects of hot water on N-nitrosomethylbenzylamine-induced esophageal tumorigenesis in F344 rats. Oncol Rep. 2003;10:421–6. https://doi.org/10.3892/or.10.2.421. 12579283

[35] Houessou JK, Maloug S, Leveque AS, Delteil C, Heyd B, Camel V (2007). Effect of roasting conditions on the polycyclic aromatic hydrocarbon content in ground Arabica coffee and coffee brew. J Agric Food Chem.

[36] Kamangar F, Schantz MM, Abnet CC, Fagundes RB, Dawsey SM (2008). High levels of carcinogenic polycyclic aromatic hydrocarbons in mate drinks. Cancer Epidemiol Biomark Prev.

[37] Vieira MA, Maraschin M, Rovaris ÂA, De RD, Amboni MC, Pagliosa CM, et al. Occurrence of polycyclic aromatic hydrocarbons throughout the processing stages of erva-mate (*Ilex Paraguariensis*). Food Addit Contam. 2010;27:776–82. 10.1080/19440041003587310 20349373

[38] Kowalski BJ, Rigdon A, Cochran J. Analytical method for polycyclic aromatic hydrocarbons (PAHs) in yerba mate tea using modified QuEChERS, solid phase extraction and GC-TOFMS and GC-MS/MS. 2015. https://www.restek.com/pdfs/FFAN2086-UNV.pdf.

[39] Golozar A, Fagundes RB, Etemadi A, Schantz MM, Kamangar F, Abnet CC (2012). Significant variation in the concentration of carcinogenic polycyclic aromatic hydrocarbons in yerba mate samples by brand, batch, and processing method. Environ Sci Technol.

[40] Winkler G, Damaty M, El SC, Lachenmeier DW. Mate - A “ new ” caffeinated raw material for the food and beverage industry. Ernahrungs Umschau. 2015;61:160–3. https://doi.org/10.4455/eu.2014.027.

[41] Tfouni SAV, Serrate CS, Leme FM, Camargo MCR, Teles CRA, Cipolli KMVAB, et al. Polycyclic aromatic hydrocarbons in coffee brew: influence of roasting and brewing procedures in two *Coffea* cultivars. LWT Food Sci Technol. 2013;50:526–30. https://doi.org/10.1016/j.lwt.2012.08.015.

[42] Sadowska-Rociek A, Surma M, Cieślik E (2015). Determination of polycyclic aromatic hydrocarbons in coffee and coffee substitutes using dispersive SPE and gas chromatography-mass spectrometry. Food Anal Methods.

[43] Perrone D, Rajoy RRS, Farah A (2010). Effect of coffee roasting on the formation of polycyclic aromatic hydrocarbons.

[44] Badolato ESG, Martins MS, Aued-Pimentel S, Alaburda J, Kumagai EE, Baptista GG (2006). Sistematic study of benzo[a]pyrene in coffee samples. J Braz Chem Soc.

[45] Moreira DP. Efeito da torrefação sobre aspectos benéficos e prejudiciais do café à saúde Humana: uma abordagem holística. DSc Thesis. Universidade Federal do Rio de Janeiro, Chemistry Institute, Rio de Janeiro, Brazil. 2009. p. 190.

[46] Zuin VG, Montero L, Bauer C, Popp P (2005). Stir bar sorptive extraction and high-performance liquid chromatography-fluorescence detection for the determination of polycyclic aromatic hydrocarbons in mate teas. J Chromatogr A.

[47] Viñas P, Campillo N, Aguinaga N, Pérez-Cánovas E, Hernández-Córdoba M (2007). Use of headspace solid-phase microextraction coupled to liquid chromatography for the analysis of polycyclic aromatic hydrocarbons in tea infusions. J Chromatogr A.

[48] Adisa A, Jimenez A, Woodham C, Anthony K, Saleh MA (2016). Determination of polycyclic aromatic hydrocarbons in dry tea. J Environ Sci Heal.

[49] Duedahl-Olesen L, Navaratnam MA, Jewula J, Jensen AH (2014). PAH in some Brands of tea and Coffee. Polycycl Aromat Compd.

[50] Ishizaki A, Saito K, Hanioka N, Narimatsu S, Kataoka H (2010). Determination of polycyclic aromatic hydrocarbons in food samples by automated on-line in-tube solid-phase microextraction coupled with high-performance liquid chromatography-fluorescence detection. J Chromatogr A.

[51] Pissinatti R, Nunes CM, de Souza AG, Junqueira RG, de Souza SVC (2015). Simultaneous analysis of 10 polycyclic aromatic hydrocarbons in roasted coffee by isotope dilution gas chromatography-mass spectrometry: optimization, in-house method validation and application to an exploratory study. Food Control.

[52] Sadowska-Rociek A, Surma M (2014). Comparison of different modifications on QuEChERS sample preparation method for PAHs determination in black, green, red and white tea. Env Sci Pollut Res.

[53] Bishnoi NR, Mehta U, Umashanker Sain GGP (2005). Quantification of polycyclic aromatic hydrocarbons in tea and coffee samples of Mumbai city (India) by high performance liquid chromatography. Environ Monit Assess.

[54] Schlemitz S, Pfannhauser W (1996). Analysis of nitro-PAHs in food matrices by on-line reduction and high performance liquid chromatography. Food Addit Contam.

[55] Pincemaille J, Schummer C, Heinen E, Determination MG (2013). Of polycyclic aromatic hydrocarbons in smoked and non-smoked black teas and tea infusions. Food Chem.

[56] Lin D, Zhu L (2004). Polycyclic aromatic hydrocarbons: pollution and source analysis of a black tea. J Agric Food Chem.

[57] Farah A. Coffee constituents. In: Chu Y, editor Coffee: Emerging Health Effects and Disease Prevention IFT Press/Wiley-Blackwell; 2012. p. 21. https://doi.org/10.1002/9781119949893.ch2.

[58] Kayali-Sayadi MN, Rubio-Barroso S, Cuesta-Jimenez MP, Polo-Díez LM (1999). A new method for the determination of selected PAHs in coffee brew samples by HPLC with fluorimetric detection and solid-phase extraction. J Liq Chromatogr Relat Technol.

[59] Ziegenhals K, Jira W, Speer K (2008). Polycyclic aromatic hydrocarbons (PAH) in various types of tea. Eur Food Res Technol.

[60] Rehm J, Lachenmeier DW, Room R (2014). Why does society accept a higher risk for alcohol than for other voluntary or involuntary risks?. BMC Med.

[61] Houessou JK, Benac C, Delteil C, Camel V (2005). Determination of polycyclic aromatic hydrocarbons in coffee brew using solid-phase extraction. J Agric Food Chem.

[62] Grover IS, Sharma R, Singh S, Pal B (2013). Polycyclic aromatic hydrocarbons in some grounded coffee brands. Environ Monit Assess.

[63] Iwegbue CMA, Agadaga H, Bassey FI, Overah LC, Tesi GO, Nwajei GE (2015). Concentrations and profiles of polycyclic aromatic hydrocarbons in some commercial Brands of tea-, coffee-, and cocoa-based food drinks in Nigeria. Int J Food Prop.

[64] Stanciu G, Birghila S, Dobrinas S (2008). Residues of polycyclic aromatic hydrocarbons in different types of coffee. Sci Study Res.

[65] Hpas A, Dieta NA, População DA, Cristiane M, De Camargo R, Cecília M (2002). Chá-Mate e Café Como Fontes de Hidrocarbonetos Policíclicos. Ciênc Tecnol Aliment.

[66] Lee K, Shin H (2010). Determination of polycyclic aromatic hydrocarbons in commercial roasted coffee beans. Food Sci Biotechnol.

[67] Jimenez A, Adisa A, Woodham C, Saleh M. Determination of polycyclic aromatic hydrocarbons in roasted coffee. J Environ Sci Health B. 2014;49:828–35. https://doi.org/10.1080/03601234.2014.938552. 10.1080/03601234.2014.938552 PMC4933506 25190557

[68] Schulz CM, Fritz H, Ruthenschrör A (2014). Occurrence of 15 + 1 EU priority polycyclic aromatic hydrocarbons (PAH) in various types of tea (Camellia Sinensis) and herbal infusions. Food Addit Contam.

[69] Lin D, Tu Y, Zhu L (2005). Concentrations and health risk of polycyclic aromatic hydrocarbons in tea. Food Chem Toxicol.

[70] Singh S, Vashishth A (2011). PAHs in some brands of tea. Environ Monit Assess.

[71] Martena MJ, Grutters MMP, De Groot HNKE, Monitoring RI (2011). Of polycyclic aromatic hydrocarbons (PAH) in food supplements containing botanicals and other ingredients on the Dutch market. Food Addit Contam.

[72] Shi Y, Wu H, Wang C, Guo X, Du J, Du L (2016). Determination of polycyclic aromatic hydrocarbons in coffee and tea samples by magnetic solid-phase extraction coupled with HPLC-FLD. Food Chem.

[73] Fiedler H (2002). PCDD/PCDF, chlorinated pesticides and PAH in Chinese teas. Chemosphere.

[74] Khiadani M, Amin MM, Beik FM, Ebrahimi A, Farhadkhani M, Mohammadi-Moghadam F. Determination of polycyclic aromatic hydrocarbons concentration in eight brands of black tea which are used more in Iran. Int J Environ Health Eng. 2013;2:40. https://doi.org/10.4103/2277-9183.122427.

[75] Garcia Londoño VA, Reynoso CM, Resnik SL (2015). Polycyclic aromatic hydrocarbons (PAHs) survey on tea (Camellia Sinensis) commercialized in Argentina. Food Control.

[76] Drabova L, Pulkrabova J, Kalachova K, Tomaniova M, Kocourek V, Hajslova J (2012). Rapid determination of polycyclic aromatic hydrocarbons (PAHs ) in tea using two-dimensional gas chromatography coupled with time of flight mass spectrometry. Talanta.

[77] Li X, Li N, Luo H, Lin L, Zou Z, Jia Y (2011). A novel synchronous fluorescence spectroscopic approach for the rapid determination of three polycyclic aromatic hydrocarbons in tea with simple microwave-assisted pretreatment of sample. J Agric Food Chem.

[78] Kayali-Sayadi MN, Rubio-Barroso S, Cuesta-Jimenez MP, Polo-Díez LM (1998). Rapid determination of polycyclic aromatic hydrocarbons in tea infusion samples by high-performance liquid chromatography and fluorimetric detection based on solid-phase extraction. Analyst.

[79] De KN, Ton S, Stegen GHD (1987). Rapid determination of benzoaIpyrene in roasted coffee and coffee brew by high-performance liquid chromatography with fluorescence detection. J Agric Food Chem.

[80] Mohammadi-Moghadam F, Amin M, Beik F, Ebrahimi A, Farhadkhani M, Khiadani (Hajian) M (2013). Determination of polycyclic aromatic hydrocarbons concentration in eight brands of black tea which are used more in Iran. Int J Environ Health Eng.

[81] Maier HG (1991). Carcinogenic compound content in coffee beans. Café Cacao Thé.

