# Supplementary material for: Comparative oesophageal cancer risk assessment of hot beverage consumption (coffee, mate and tea): the margin of exposure of PAH vs very hot temperatures
Source: BMC Cancer. 2018 Mar 1;18:236. doi: 10.1186/s12885-018-4060-z (PMC5831222; doi:10.1186/s12885-018-4060-z)
Supplement: Supplementary file 1 — Supplementary Tables S1-S3. Supplementary Table S1. Distribution functions as input for probabilistic analysis; Supplementary Table S2. Detailed calculation methodology for probabilistic risk assessment of PAH due to beverage consumption; Supplementary Table S3. Raw results of probabilistic estimation of BaP and PAH4 exposure and margin of exposure (MOE) using 10,000 iterations. (DOC 250 kb) [file 12885_2018_4060_MOESM1_ESM.doc]

Supplementary Table S1. Distribution functions as input for probabilistic analysis

| **Parameter** | **Risk function ^a^** | **Unit** |
| --- | --- | --- |
| Bodyweight according to Lachenmeier and Rehm (2015) [27] | RiskNormal(73.9;12) | kg |
| Extraction % (i.e. the percent amount of PAH transferred from the content in the leaves or beans into the final beverage infusion) | Average of the following risk functions: RiskUniform(6;37) for BaP in mate; 50% for PAH4 in mate; RiskUniform(2.3;7.7) for BaP in tea; RiskUniform(11;100) for PAH4 in tea; RiskUniform(0.8;35) for BaP in coffee; RiskUniform(13;14) for PAH4 in coffee. The resulting overall distribution for extraction % was: average 35%, P5: 27%, P50: 27% and P95: 35% ^b^ | % |
| Leaves/beans per L (amount used for preparation of beverages necessary for calculation from leaves/beans to the final beverage infusion) | Mate: 20-50 g/L (own estimation based on [6,12,31]): RiskUniform(20;50)  Tea: 10-20 g/L (own estimation including ISO 3103:1980 [29]): RiskUniform(10;20)  Coffee: 40-90 g/L (own estimation including ISO 6668:2008 [30]): RiskUniform(40;90) | g/L |
| Per capita consumption (of leaves/beans) | Mate: RiskUniform(1.2;7.8) based on data from Brazil/Argentina/Paraguay/Uruguay [1]  Tea: RiskUniform(1;7.54) based on data from tea consuming countries [1]  Coffee: RiskExtvalue(7.92548;0.94944) based on data from coffee consuming countries [1] | kg/person/year |
| Risk fit distribution for occurrence data of PAH in leaves/beans or infusions | In the majority of cases (>90%), the “Expon” function was found as best fitting the datasets. For reasons of consistency all datasets were fitted with the following function: RiskFitDistribution(Data;”Expon”;”AIC”;0;”INF”) | µg/kg (leaves/beans) or µg/L (infusions) |
| Recalculation to content in final beverage infusions for datasets which provided analytical data of leaves or beans | Leaves/Beans (g/L) / 1000 * Distribution function (µg/kg in leaves/beans) * Extraction % / 100 | µg/L |
| Percentage of consumers in population | 80 ^c^ | % |
| BMDL(BaP) | 70 ^c^ | µg/kg bw/day |
| BMDL(PAH4) | 340 ^c^ | µg/kg bw/day |

^a^ RiskNormal(mean;standard deviation) specifies a normal distribution with the entered mean and standard deviation. RiskUniform(minimum;maximum) specifies a uniform probability distribution with the entered minimum and maximum values. Every value across the range has an equal likelihood of occurrence (“no knowledge” distribution). RiskExtvalue(alpha;beta) specifies an extreme value distribution with the position parameters alpha and beta. RiskExpon(beta) specifies an exponential distribution with a beta value. The average of the distribution is similar to beta.

^b^ The extraction percentages were obtained from the following references: 6-37% BaP mate[35,56], 50% PAH4 mate [35,69], 2.3-7.7 tea [80,50], 11-100 PAH tea [49,55,68,80], 0.8-35 BaP coffee [79,81] , 13-14 PAH coffee [34]. Due to the high variation between the different literature studies from extraction in the low percentages up to 100%, which are not completely plausible. One single overall distribution of all the studies was used for the calculations.

^c^ calculation as point estimate (no distribution available)

**Supplementary Table S2. Detailed calculation methodology for probabilistic risk assessment of PAH due to beverage consumption**

| **Parameter** | **Calculation formula** **for the software package @Risk for Excel Version 7.5.0 (Palisade, Corporation, Ithaca, NY, USA)** | **Unit** |
| --- | --- | --- |
| Individual Exposure for data based on leaves/beans analysis | = Recalculation to content in final beverage infusions (µg/L) / Risk function for bodyweight (kg) * Daily consumption (L/day) | µg/kg bw/day |
| Individual Exposure for data based on final beverage infusions analysis | = Risk fit distribution of dataset (µg/L) / Risk function for bodyweight (kg) * Daily consumption (L/day) | µg/kg bw/day |
| Per capita exposure | = Per capita consumption (kg) * Percentage of consumers in population (%) / 100 / 365 days * Risk fit distribution of leaves/beans dataset (µg/kg) / Risk function for bodyweight (kg) * Extraction (%) / 100 | µg/kg bw/day |
| MOE | = BMDL (µg/kg bw/day) / Exposure (µg/kg bw/day) | - |

**Supplementary Table S3. Raw results of probabilistic estimation of BaP and PAH4 exposure and margin of exposure (MOE) using 10,000 iterations**

| **Name** | **Minimum** | **Maximum** | **Mean** | **Std Deviation** | **5% Perc** | **25% Perc** | **50% Perc** | **75% Perc** | **90% Perc** | **95% Perc** |
| --- | --- | --- | --- | --- | --- | --- | --- | --- | --- | --- |
| A Exposure 1 cup 0.2 L (µg/kg bw/day) / 01 BaP-LOD-Mate-Leaves | 4.34E-08 | 2.14E-02 | 1.94E-03 | 2.14E-03 | 9.27E-05 | 5.14E-04 | 1.24E-03 | 2.61E-03 | 4.49E-03 | 6.10E-03 |
| A Exposure 1 cup 0.2 L (µg/kg bw/day) / 02 PAH4-LOD-Mate-Leaves | 3.98E-08 | 1.57E-01 | 1.11E-02 | 1.23E-02 | 4.97E-04 | 2.91E-03 | 7.12E-03 | 1.50E-02 | 2.57E-02 | 3.47E-02 |
| A Exposure 1 cup 0.2 L (µg/kg bw/day) / 03 BaP-LOD-Mate-Infusion | 2.53E-08 | 3.26E-03 | 2.85E-04 | 2.92E-04 | 1.40E-05 | 7.99E-05 | 1.94E-04 | 3.89E-04 | 6.63E-04 | 8.62E-04 |
| A Exposure 1 cup 0.2 L (µg/kg bw/day) / 04 PAH4-LOD-Mate-Infusion | 1.24E-08 | 7.38E-03 | 7.22E-04 | 7.50E-04 | 3.64E-05 | 2.01E-04 | 4.93E-04 | 9.79E-04 | 1.65E-03 | 2.18E-03 |
| A Exposure 1 cup 0.2 L (µg/kg bw/day) / 05 BaP-LOD-Tea-Leaves | 1.31E-08 | 2.26E-03 | 1.65E-04 | 1.82E-04 | 7.81E-06 | 4.30E-05 | 1.07E-04 | 2.21E-04 | 3.85E-04 | 5.12E-04 |
| A Exposure 1 cup 0.2 L (µg/kg bw/day) / 06 PAH4-LOD-Tea-Leaves | 9.22E-08 | 1.35E-02 | 1.02E-03 | 1.10E-03 | 4.74E-05 | 2.73E-04 | 6.67E-04 | 1.38E-03 | 2.41E-03 | 3.26E-03 |
| A Exposure 1 cup 0.2 L (µg/kg bw/day) / 09 BaP-LOD-Coffee-Beans | 6.20E-09 | 3.26E-03 | 1.88E-04 | 2.09E-04 | 8.76E-06 | 4.90E-05 | 1.21E-04 | 2.52E-04 | 4.43E-04 | 5.95E-04 |
| A Exposure 1 cup 0.2 L (µg/kg bw/day) / 10 PAH4-LOD-Coffee Beans | 7.28E-08 | 3.17E-02 | 1.85E-03 | 2.05E-03 | 8.33E-05 | 4.85E-04 | 1.20E-03 | 2.48E-03 | 4.34E-03 | 5.77E-03 |
| A Exposure 1 cup 0.2 L (µg/kg bw/day) / 11 BaP-LOD-Coffee-Infusion | 8.19E-09 | 2.40E-03 | 1.71E-04 | 1.76E-04 | 8.52E-06 | 4.81E-05 | 1.16E-04 | 2.35E-04 | 3.92E-04 | 5.21E-04 |
| A Exposure 1 cup 0.2 L (µg/kg bw/day) / 12 PAH4-LOD-Coffee-Infusion | 1.80E-08 | 9.10E-03 | 1.00E-03 | 1.04E-03 | 5.06E-05 | 2.82E-04 | 6.83E-04 | 1.38E-03 | 2.30E-03 | 3.10E-03 |
| B Exposure 2 cups 0.4 L (µg/kg bw/day) / 01 BaP-LOD-Mate-Leaves | 8.69E-08 | 4.27E-02 | 3.87E-03 | 4.29E-03 | 1.85E-04 | 1.03E-03 | 2.47E-03 | 5.22E-03 | 8.99E-03 | 1.22E-02 |
| B Exposure 2 cups 0.4 L (µg/kg bw/day) / 02 PAH4-LOD-Mate-Leaves | 7.96E-08 | 3.15E-01 | 2.22E-02 | 2.46E-02 | 9.95E-04 | 5.81E-03 | 1.42E-02 | 3.00E-02 | 5.15E-02 | 6.94E-02 |
| B Exposure 2 cups 0.4 L (µg/kg bw/day) / 03 BaP-LOD-Mate-Infusion | 5.06E-08 | 6.52E-03 | 5.70E-04 | 5.84E-04 | 2.80E-05 | 1.60E-04 | 3.88E-04 | 7.78E-04 | 1.33E-03 | 1.72E-03 |
| B Exposure 2 cups 0.4 L (µg/kg bw/day) / 04 PAH4-LOD-Mate-Infusion | 2.47E-08 | 1.48E-02 | 1.44E-03 | 1.50E-03 | 7.28E-05 | 4.02E-04 | 9.86E-04 | 1.96E-03 | 3.31E-03 | 4.35E-03 |
| B Exposure 2 cups 0.4 L (µg/kg bw/day) / 05 BaP-LOD-Tea-Leaves | 2.62E-08 | 4.53E-03 | 3.30E-04 | 3.64E-04 | 1.56E-05 | 8.60E-05 | 2.13E-04 | 4.41E-04 | 7.71E-04 | 1.02E-03 |
| B Exposure 2 cups 0.4 L (µg/kg bw/day) / 06 PAH4-LOD-Tea-Leaves | 1.84E-07 | 2.69E-02 | 2.04E-03 | 2.21E-03 | 9.47E-05 | 5.46E-04 | 1.33E-03 | 2.76E-03 | 4.81E-03 | 6.52E-03 |
| B Exposure 2 cups 0.4 L (µg/kg bw/day) / 09 BaP-LOD-Coffee-Beans | 1.24E-08 | 6.52E-03 | 3.77E-04 | 4.17E-04 | 1.75E-05 | 9.80E-05 | 2.43E-04 | 5.05E-04 | 8.86E-04 | 1.19E-03 |
| B Exposure 2 cups 0.4 L (µg/kg bw/day) / 10 PAH4-LOD-Coffee Beans | 1.46E-07 | 6.34E-02 | 3.70E-03 | 4.10E-03 | 1.67E-04 | 9.71E-04 | 2.40E-03 | 4.95E-03 | 8.69E-03 | 1.15E-02 |
| B Exposure 2 cups 0.4 L (µg/kg bw/day) / 11 BaP-LOD-Coffee-Infusion | 1.64E-08 | 4.81E-03 | 3.41E-04 | 3.51E-04 | 1.70E-05 | 9.61E-05 | 2.33E-04 | 4.70E-04 | 7.84E-04 | 1.04E-03 |
| B Exposure 2 cups 0.4 L (µg/kg bw/day) / 12 PAH4-LOD-Coffee-Infusion | 3.60E-08 | 1.82E-02 | 2.01E-03 | 2.08E-03 | 1.01E-04 | 5.63E-04 | 1.37E-03 | 2.77E-03 | 4.59E-03 | 6.20E-03 |
| C Exposure 1 L (µg/kg bw/day) / 01 BaP-LOD-Mate-Leaves | 2.17E-07 | 1.07E-01 | 9.68E-03 | 1.07E-02 | 4.64E-04 | 2.57E-03 | 6.19E-03 | 1.30E-02 | 2.25E-02 | 3.05E-02 |
| C Exposure 1 L (µg/kg bw/day) / 02 PAH4-LOD-Mate-Leaves | 1.99E-07 | 7.87E-01 | 5.54E-02 | 6.14E-02 | 2.49E-03 | 1.45E-02 | 3.56E-02 | 7.49E-02 | 1.29E-01 | 1.73E-01 |
| C Exposure 1 L (µg/kg bw/day) / 03 BaP-LOD-Mate-Infusion | 1.27E-07 | 1.63E-02 | 1.42E-03 | 1.46E-03 | 7.00E-05 | 3.99E-04 | 9.71E-04 | 1.95E-03 | 3.32E-03 | 4.31E-03 |
| C Exposure 1 L (µg/kg bw/day) / 04 PAH4-LOD-Mate-Infusion | 6.18E-08 | 3.69E-02 | 3.61E-03 | 3.75E-03 | 1.82E-04 | 1.01E-03 | 2.47E-03 | 4.90E-03 | 8.26E-03 | 1.09E-02 |
| C Exposure 1 L (µg/kg bw/day) / 05 BaP-LOD-Tea-Leaves | 6.56E-08 | 1.13E-02 | 8.26E-04 | 9.10E-04 | 3.90E-05 | 2.15E-04 | 5.33E-04 | 1.10E-03 | 1.93E-03 | 2.56E-03 |
| C Exposure 1 L (µg/kg bw/day) / 06 PAH4-LOD-Tea-Leaves | 4.61E-07 | 6.73E-02 | 5.11E-03 | 5.51E-03 | 2.37E-04 | 1.36E-03 | 3.33E-03 | 6.90E-03 | 1.20E-02 | 1.63E-02 |
| C Exposure 1 L (µg/kg bw/day) / 09 BaP-LOD-Coffee-Beans | 3.10E-08 | 1.63E-02 | 9.42E-04 | 1.04E-03 | 4.38E-05 | 2.45E-04 | 6.06E-04 | 1.26E-03 | 2.22E-03 | 2.97E-03 |
| C Exposure 1 L (µg/kg bw/day) / 10 PAH4-LOD-Coffee Beans | 3.64E-07 | 1.59E-01 | 9.26E-03 | 1.02E-02 | 4.17E-04 | 2.43E-03 | 6.00E-03 | 1.24E-02 | 2.17E-02 | 2.89E-02 |
| C Exposure 1 L (µg/kg bw/day) / 11 BaP-LOD-Coffee-Infusion | 4.10E-08 | 1.20E-02 | 8.54E-04 | 8.79E-04 | 4.26E-05 | 2.40E-04 | 5.81E-04 | 1.17E-03 | 1.96E-03 | 2.61E-03 |
| C Exposure 1 L (µg/kg bw/day) / 12 PAH4-LOD-Coffee-Infusion | 8.99E-08 | 4.55E-02 | 5.02E-03 | 5.19E-03 | 2.53E-04 | 1.41E-03 | 3.42E-03 | 6.92E-03 | 1.15E-02 | 1.55E-02 |
| D Exposure 3 L (µg/kg bw/day) / 01 BaP-LOD-Mate-Leaves | 6.52E-07 | 3.21E-01 | 2.90E-02 | 3.22E-02 | 1.39E-03 | 7.71E-03 | 1.86E-02 | 3.91E-02 | 6.74E-02 | 9.15E-02 |
| D Exposure 3 L (µg/kg bw/day) / 02 PAH4-LOD-Mate-Leaves | 5.97E-07 | 2.36E+00 | 1.66E-01 | 1.84E-01 | 7.46E-03 | 4.36E-02 | 1.07E-01 | 2.25E-01 | 3.86E-01 | 5.20E-01 |
| D Exposure 3 L (µg/kg bw/day) / 03 BaP-LOD-Mate-Infusion | 3.80E-07 | 4.89E-02 | 4.27E-03 | 4.38E-03 | 2.10E-04 | 1.20E-03 | 2.91E-03 | 5.84E-03 | 9.95E-03 | 1.29E-02 |
| D Exposure 3 L (µg/kg bw/day) / 04 PAH4-LOD-Mate-Infusion | 1.85E-07 | 1.11E-01 | 1.08E-02 | 1.13E-02 | 5.46E-04 | 3.02E-03 | 7.40E-03 | 1.47E-02 | 2.48E-02 | 3.26E-02 |
| D Exposure 3 L (µg/kg bw/day) / 05 BaP-LOD-Tea-Leaves | 1.97E-07 | 3.39E-02 | 2.48E-03 | 2.73E-03 | 1.17E-04 | 6.45E-04 | 1.60E-03 | 3.31E-03 | 5.78E-03 | 7.68E-03 |
| D Exposure 3 L (µg/kg bw/day) / 06 PAH4-LOD-Tea-Leaves | 1.38E-06 | 2.02E-01 | 1.53E-02 | 1.65E-02 | 7.11E-04 | 4.09E-03 | 1.00E-02 | 2.07E-02 | 3.61E-02 | 4.89E-02 |
| D Exposure 3 L (µg/kg bw/day) / 09 BaP-LOD-Coffee-Beans | 9.31E-08 | 4.89E-02 | 2.83E-03 | 3.13E-03 | 1.31E-04 | 7.35E-04 | 1.82E-03 | 3.79E-03 | 6.65E-03 | 8.92E-03 |
| D Exposure 3 L (µg/kg bw/day) / 10 PAH4-LOD-Coffee Beans | 1.09E-06 | 4.76E-01 | 2.78E-02 | 3.07E-02 | 1.25E-03 | 7.28E-03 | 1.80E-02 | 3.71E-02 | 6.52E-02 | 8.66E-02 |
| D Exposure 3 L (µg/kg bw/day) / 11 BaP-LOD-Coffee-Infusion | 1.23E-07 | 3.60E-02 | 2.56E-03 | 2.64E-03 | 1.28E-04 | 7.21E-04 | 1.74E-03 | 3.52E-03 | 5.88E-03 | 7.82E-03 |
| D Exposure 3 L (µg/kg bw/day) / 12 PAH4-LOD-Coffee-Infusion | 2.70E-07 | 1.36E-01 | 1.51E-02 | 1.56E-02 | 7.59E-04 | 4.22E-03 | 1.02E-02 | 2.08E-02 | 3.44E-02 | 4.65E-02 |
| A MOE 1 cup 0.2 L / 01 BaP-LOD-Mate-Leaves | 3.28E+03 | 1.61E+09 | 4.97E+05 | 1.65E+07 | 1.15E+04 | 2.68E+04 | 5.66E+04 | 1.36E+05 | 3.83E+05 | 7.54E+05 |
| A MOE 1 cup 0.2 L / 02 PAH4-LOD-Mate-Leaves | 2.16E+03 | 8.54E+09 | 1.13E+06 | 8.54E+07 | 9.78E+03 | 2.27E+04 | 4.77E+04 | 1.17E+05 | 3.30E+05 | 6.83E+05 |
| A MOE 1 cup 0.2 L / 03 BaP-LOD-Mate-Infusion | 2.15E+04 | 2.76E+09 | 2.45E+06 | 3.69E+07 | 8.11E+04 | 1.80E+05 | 3.61E+05 | 8.76E+05 | 2.38E+06 | 4.97E+06 |
| A MOE 1 cup 0.2 L / 04 PAH4-LOD-Mate-Infusion | 4.60E+04 | 2.75E+10 | 6.85E+06 | 2.79E+08 | 1.56E+05 | 3.47E+05 | 6.89E+05 | 1.69E+06 | 4.61E+06 | 9.34E+06 |
| A MOE 1 cup 0.2 L / 05 BaP-LOD-Tea-Leaves | 3.09E+04 | 5.34E+09 | 4.50E+06 | 7.06E+07 | 1.37E+05 | 3.17E+05 | 6.57E+05 | 1.63E+06 | 4.39E+06 | 8.96E+06 |
| A MOE 1 cup 0.2 L / 06 PAH4-LOD-Tea-Leaves | 2.53E+04 | 3.69E+09 | 3.35E+06 | 5.09E+07 | 1.04E+05 | 2.46E+05 | 5.10E+05 | 1.25E+06 | 3.55E+06 | 7.15E+06 |
| A MOE 1 cup 0.2 L / 09 BaP-LOD-Coffee-Beans | 2.15E+04 | 1.13E+10 | 4.48E+06 | 1.16E+08 | 1.18E+05 | 2.77E+05 | 5.77E+05 | 1.43E+06 | 3.90E+06 | 7.98E+06 |
| A MOE 1 cup 0.2 L / 10 PAH4-LOD-Coffee Beans | 1.07E+04 | 4.67E+09 | 2.18E+06 | 5.00E+07 | 5.88E+04 | 1.37E+05 | 2.83E+05 | 7.00E+05 | 1.91E+06 | 4.07E+06 |
| A MOE 1 cup 0.2 L / 11 BaP-LOD-Coffee-Infusion | 2.91E+04 | 8.54E+09 | 4.61E+06 | 9.83E+07 | 1.34E+05 | 2.98E+05 | 6.02E+05 | 1.46E+06 | 4.01E+06 | 8.21E+06 |
| A MOE 1 cup 0.2 L / 12 PAH4-LOD-Coffee-Infusion | 3.74E+04 | 1.89E+10 | 5.01E+06 | 1.94E+08 | 1.10E+05 | 2.46E+05 | 4.97E+05 | 1.21E+06 | 3.29E+06 | 6.72E+06 |
| B MOE 2 cups 0.4 L / 01 BaP-LOD-Mate-Leaves | 1.64E+03 | 8.06E+08 | 2.49E+05 | 8.27E+06 | 5.74E+03 | 1.34E+04 | 2.83E+04 | 6.81E+04 | 1.91E+05 | 3.77E+05 |
| B MOE 2 cups 0.4 L / 02 PAH4-LOD-Mate-Leaves | 1.08E+03 | 4.27E+09 | 5.67E+05 | 4.27E+07 | 4.89E+03 | 1.13E+04 | 2.39E+04 | 5.85E+04 | 1.65E+05 | 3.42E+05 |
| B MOE 2 cups 0.4 L / 03 BaP-LOD-Mate-Infusion | 1.07E+04 | 1.38E+09 | 1.22E+06 | 1.85E+07 | 4.06E+04 | 8.99E+04 | 1.80E+05 | 4.38E+05 | 1.19E+06 | 2.48E+06 |
| B MOE 2 cups 0.4 L / 04 PAH4-LOD-Mate-Infusion | 2.30E+04 | 1.38E+10 | 3.43E+06 | 1.39E+08 | 7.81E+04 | 1.73E+05 | 3.45E+05 | 8.45E+05 | 2.31E+06 | 4.67E+06 |
| B MOE 2 cups 0.4 L / 05 BaP-LOD-Tea-Leaves | 1.55E+04 | 2.67E+09 | 2.25E+06 | 3.53E+07 | 6.83E+04 | 1.59E+05 | 3.29E+05 | 8.14E+05 | 2.19E+06 | 4.48E+06 |
| B MOE 2 cups 0.4 L / 06 PAH4-LOD-Tea-Leaves | 1.26E+04 | 1.84E+09 | 1.68E+06 | 2.55E+07 | 5.21E+04 | 1.23E+05 | 2.55E+05 | 6.23E+05 | 1.77E+06 | 3.58E+06 |
| B MOE 2 cups 0.4 L / 09 BaP-LOD-Coffee-Beans | 1.07E+04 | 5.64E+09 | 2.24E+06 | 5.82E+07 | 5.88E+04 | 1.39E+05 | 2.88E+05 | 7.14E+05 | 1.95E+06 | 3.99E+06 |
| B MOE 2 cups 0.4 L / 10 PAH4-LOD-Coffee Beans | 5.36E+03 | 2.34E+09 | 1.09E+06 | 2.50E+07 | 2.94E+04 | 6.86E+04 | 1.42E+05 | 3.50E+05 | 9.53E+05 | 2.04E+06 |
| B MOE 2 cups 0.4 L / 11 BaP-LOD-Coffee-Infusion | 1.46E+04 | 4.27E+09 | 2.30E+06 | 4.92E+07 | 6.71E+04 | 1.49E+05 | 3.01E+05 | 7.28E+05 | 2.01E+06 | 4.11E+06 |
| B MOE 2 cups 0.4 L / 12 PAH4-LOD-Coffee-Infusion | 1.87E+04 | 9.45E+09 | 2.51E+06 | 9.68E+07 | 5.48E+04 | 1.23E+05 | 2.49E+05 | 6.04E+05 | 1.65E+06 | 3.36E+06 |
| C MOE 1 L / 01 BaP-LOD-Mate-Leaves | 6.55E+02 | 3.22E+08 | 9.94E+04 | 3.31E+06 | 2.30E+03 | 5.37E+03 | 1.13E+04 | 2.72E+04 | 7.65E+04 | 1.51E+05 |
| C MOE 1 L / 02 PAH4-LOD-Mate-Leaves | 4.32E+02 | 1.71E+09 | 2.27E+05 | 1.71E+07 | 1.96E+03 | 4.53E+03 | 9.55E+03 | 2.34E+04 | 6.60E+04 | 1.37E+05 |
| C MOE 1 L / 03 BaP-LOD-Mate-Infusion | 4.29E+03 | 5.53E+08 | 4.89E+05 | 7.39E+06 | 1.62E+04 | 3.60E+04 | 7.21E+04 | 1.75E+05 | 4.75E+05 | 9.93E+05 |
| C MOE 1 L / 04 PAH4-LOD-Mate-Infusion | 9.21E+03 | 5.51E+09 | 1.37E+06 | 5.58E+07 | 3.12E+04 | 6.94E+04 | 1.38E+05 | 3.38E+05 | 9.22E+05 | 1.87E+06 |
| C MOE 1 L / 05 BaP-LOD-Tea-Leaves | 6.19E+03 | 1.07E+09 | 9.00E+05 | 1.41E+07 | 2.73E+04 | 6.35E+04 | 1.31E+05 | 3.26E+05 | 8.77E+05 | 1.79E+06 |
| C MOE 1 L / 06 PAH4-LOD-Tea-Leaves | 5.05E+03 | 7.37E+08 | 6.71E+05 | 1.02E+07 | 2.09E+04 | 4.93E+04 | 1.02E+05 | 2.49E+05 | 7.10E+05 | 1.43E+06 |
| C MOE 1 L / 09 BaP-LOD-Coffee-Beans | 4.29E+03 | 2.26E+09 | 8.97E+05 | 2.33E+07 | 2.35E+04 | 5.54E+04 | 1.15E+05 | 2.86E+05 | 7.80E+05 | 1.60E+06 |
| C MOE 1 L / 10 PAH4-LOD-Coffee Beans | 2.14E+03 | 9.34E+08 | 4.35E+05 | 1.00E+07 | 1.18E+04 | 2.75E+04 | 5.67E+04 | 1.40E+05 | 3.81E+05 | 8.14E+05 |
| C MOE 1 L / 11 BaP-LOD-Coffee-Infusion | 5.83E+03 | 1.71E+09 | 9.21E+05 | 1.97E+07 | 2.68E+04 | 5.96E+04 | 1.20E+05 | 2.91E+05 | 8.02E+05 | 1.64E+06 |
| C MOE 1 L / 12 PAH4-LOD-Coffee-Infusion | 7.48E+03 | 3.78E+09 | 1.00E+06 | 3.87E+07 | 2.19E+04 | 4.91E+04 | 9.95E+04 | 2.41E+05 | 6.59E+05 | 1.34E+06 |
| D MOE 3 L / 01 BaP-LOD-Mate-Leaves | 2.18E+02 | 1.07E+08 | 3.31E+04 | 1.10E+06 | 7.65E+02 | 1.79E+03 | 3.77E+03 | 9.08E+03 | 2.55E+04 | 5.03E+04 |
| D MOE 3 L / 02 PAH4-LOD-Mate-Leaves | 1.44E+02 | 5.69E+08 | 7.56E+04 | 5.70E+06 | 6.52E+02 | 1.51E+03 | 3.18E+03 | 7.80E+03 | 2.20E+04 | 4.56E+04 |
| D MOE 3 L / 03 BaP-LOD-Mate-Infusion | 1.43E+03 | 1.84E+08 | 1.63E+05 | 2.46E+06 | 5.41E+03 | 1.20E+04 | 2.40E+04 | 5.84E+04 | 1.58E+05 | 3.31E+05 |
| D MOE 3 L / 04 PAH4-LOD-Mate-Infusion | 3.07E+03 | 1.84E+09 | 4.57E+05 | 1.86E+07 | 1.04E+04 | 2.31E+04 | 4.60E+04 | 1.13E+05 | 3.07E+05 | 6.23E+05 |
| D MOE 3 L / 05 BaP-LOD-Tea-Leaves | 2.06E+03 | 3.56E+08 | 3.00E+05 | 4.71E+06 | 9.10E+03 | 2.12E+04 | 4.38E+04 | 1.09E+05 | 2.92E+05 | 5.97E+05 |
| D MOE 3 L / 06 PAH4-LOD-Tea-Leaves | 1.68E+03 | 2.46E+08 | 2.24E+05 | 3.40E+06 | 6.95E+03 | 1.64E+04 | 3.40E+04 | 8.31E+04 | 2.37E+05 | 4.77E+05 |
| D MOE 3 L / 09 BaP-LOD-Coffee-Beans | 1.43E+03 | 7.52E+08 | 2.99E+05 | 7.76E+06 | 7.85E+03 | 1.85E+04 | 3.85E+04 | 9.52E+04 | 2.60E+05 | 5.32E+05 |
| D MOE 3 L / 10 PAH4-LOD-Coffee Beans | 7.15E+02 | 3.11E+08 | 1.45E+05 | 3.33E+06 | 3.92E+03 | 9.15E+03 | 1.89E+04 | 4.67E+04 | 1.27E+05 | 2.71E+05 |
| D MOE 3 L / 11 BaP-LOD-Coffee-Infusion | 1.94E+03 | 5.70E+08 | 3.07E+05 | 6.55E+06 | 8.94E+03 | 1.99E+04 | 4.01E+04 | 9.71E+04 | 2.67E+05 | 5.48E+05 |
| D MOE 3 L / 12 PAH4-LOD-Coffee-Infusion | 2.49E+03 | 1.26E+09 | 3.34E+05 | 1.29E+07 | 7.31E+03 | 1.64E+04 | 3.32E+04 | 8.05E+04 | 2.20E+05 | 4.48E+05 |
| E Exposure per capita [µg/kg bw/day] / 01 BaP-LOD-Mate-Leaves | 1.19E-07 | 5.49E-02 | 4.26E-03 | 5.09E-03 | 1.62E-04 | 9.61E-04 | 2.51E-03 | 5.57E-03 | 1.04E-02 | 1.44E-02 |
| E Exposure per capita [µg/kg bw/day] / 02 PAH4-LOD-Mate-Leaves | 9.81E-08 | 3.67E-01 | 2.45E-02 | 3.00E-02 | 9.06E-04 | 5.47E-03 | 1.45E-02 | 3.22E-02 | 5.89E-02 | 8.10E-02 |
| E MOE per capita / 02 PAH4-LOD-Mate-Leaves | 9.26E+02 | 3.47E+09 | 4.91E+05 | 3.47E+07 | 4.20E+03 | 1.05E+04 | 2.35E+04 | 6.21E+04 | 1.77E+05 | 3.74E+05 |
| E MOE per capita / 01 BaP-LOD-Mate-Leaves | 1.28E+03 | 5.86E+08 | 2.31E+05 | 6.04E+06 | 4.85E+03 | 1.26E+04 | 2.79E+04 | 7.28E+04 | 2.05E+05 | 4.32E+05 |
| E Exposure per capita [µg/kg bw/day] / 05 BaP-LOD-Tea-Leaves | 1.97E-08 | 1.19E-02 | 8.03E-04 | 9.97E-04 | 2.84E-05 | 1.79E-04 | 4.66E-04 | 1.03E-03 | 1.95E-03 | 2.78E-03 |
| E MOE per capita / 05 BaP-LOD-Tea-Leaves | 5.87E+03 | 3.55E+09 | 1.39E+06 | 3.73E+07 | 2.51E+04 | 6.79E+04 | 1.50E+05 | 3.91E+05 | 1.11E+06 | 2.47E+06 |
| E MOE per capita / 06 PAH4-LOD-Tea-Leaves | 4.61E+03 | 3.68E+09 | 1.13E+06 | 3.81E+07 | 2.03E+04 | 5.29E+04 | 1.18E+05 | 3.13E+05 | 8.70E+05 | 1.83E+06 |
| E Exposure per capita [µg/kg bw/day] / 06 PAH4-LOD-Tea-Leaves | 9.23E-08 | 7.37E-02 | 4.98E-03 | 6.19E-03 | 1.86E-04 | 1.09E-03 | 2.88E-03 | 6.42E-03 | 1.22E-02 | 1.68E-02 |
| E Exposure per capita [µg/kg bw/day] / 09 BaP-LOD-Coffe-Beans | 1.47E-08 | 5.72E-03 | 4.21E-04 | 4.53E-04 | 2.01E-05 | 1.14E-04 | 2.78E-04 | 5.70E-04 | 9.79E-04 | 1.32E-03 |
| E MOE per capita / 09 BaP-LOD-Coffee-Beans | 1.22E+04 | 4.75E+09 | 1.97E+06 | 4.96E+07 | 5.28E+04 | 1.23E+05 | 2.52E+05 | 6.12E+05 | 1.69E+06 | 3.48E+06 |
| E Exposure per capita [µg/kg bw/day] / 10 PAH4-LOD-Coffee Beans | 1.39E-07 | 7.24E-02 | 4.15E-03 | 4.51E-03 | 1.97E-04 | 1.12E-03 | 2.73E-03 | 5.57E-03 | 9.72E-03 | 1.28E-02 |
| E MOE per capita / 10 PAH4-LOD-Coffee Beans | 4.70E+03 | 2.45E+09 | 9.70E+05 | 2.53E+07 | 2.65E+04 | 6.11E+04 | 1.25E+05 | 3.03E+05 | 8.26E+05 | 1.72E+06 |
| X Recalculation Leaves/Beans to Beverage (µg/L) / 01 BaP-LOD-Mate-Leaves | 1.35E-05 | 7.96E+00 | 6.96E-01 | 7.51E-01 | 3.37E-02 | 1.87E-01 | 4.55E-01 | 9.49E-01 | 1.63E+00 | 2.18E+00 |
| X Recalculation Leaves/Beans to Beverage (µg/L) / 02 PAH4-LOD-Mate-Leaves | 1.55E-05 | 4.88E+01 | 3.99E+00 | 4.32E+00 | 1.81E-01 | 1.06E+00 | 2.61E+00 | 5.43E+00 | 9.25E+00 | 1.23E+01 |
| X Recalculation Leaves/Beans to Beverage (µg/L) / 05 BaP-LOD-Tea-Leaves | 4.50E-06 | 7.08E-01 | 5.93E-02 | 6.36E-02 | 2.94E-03 | 1.59E-02 | 3.89E-02 | 8.03E-02 | 1.38E-01 | 1.83E-01 |
| X Recalculation Leaves/Beans to Beverage (µg/L) / 06 PAH4-LOD-Tea-Leaves | 3.42E-05 | 4.89E+00 | 3.68E-01 | 3.92E-01 | 1.74E-02 | 1.00E-01 | 2.41E-01 | 5.05E-01 | 8.61E-01 | 1.13E+00 |
| X Recalculation Leaves/Beans to Beverage (µg/L) / 09 BaP-LOD-Coffee-Beans | 1.91E-06 | 1.14E+00 | 6.76E-02 | 7.27E-02 | 3.21E-03 | 1.81E-02 | 4.44E-02 | 9.22E-02 | 1.59E-01 | 2.09E-01 |
| X Recalculation Leaves/Beans to Beverage (µg/L) / 10 PAH4-LOD-Coffee Beans | 2.97E-05 | 8.25E+00 | 6.65E-01 | 7.16E-01 | 3.21E-02 | 1.80E-01 | 4.39E-01 | 8.99E-01 | 1.55E+00 | 2.08E+00 |
| Extraction % overall | 1.52E+01 | 3.97E+01 | 2.72E+01 | 4.78E+00 | 1.96E+01 | 2.36E+01 | 2.72E+01 | 3.09E+01 | 3.36E+01 | 3.50E+01 |
