# Supplementary material for: Comparative oesophageal cancer risk assessment of hot beverage consumption (coffee, mate and tea): the margin of exposure of PAH vs very hot temperatures
Source: BMC Cancer. 2018 Mar 1;18:236. doi: 10.1186/s12885-018-4060-z (PMC5831222; doi:10.1186/s12885-018-4060-z)
Supplement: Supplementary file 2 — Data appendix with raw result for benchmark dose-response modelling. Supplementary Data S1. BMD modelling for mean number of tumours per rat from Li et al. (2003); Supplementary Data S2. BMD modelling for mean volume of tumours (mm3) from Li et al. (2003). (DOC 81 kb) [file 12885_2018_4060_MOESM2_ESM.doc]

## Data appendix with raw results for benchmark dose-response modelling

**Supplementary Data S1. BMD modelling for mean number of tumours per rat from Li et al. (2003) [34]: raw data from Table II, groups 4,5,6. The temperature for the control group was not stated and assumed as being 22°C (average room temperature for testing of chemicals according to OECD guideline).**

====================================================================

Power Model. (Version: 2.18; Date: 05/19/2014)

BMDS Model Run

~~~~~~~~~~~~~~~~~~~~~~~~~~~~~~~~~~~~~~~~~~~~~~~~~~~~~~~~~~~~~~~~~~~~~

The form of the response function is:

Y[dose] = control + slope * dose^power

Dependent variable = Mean_tumors

Independent variable = Temperature

rho is set to 0

The power is restricted to be greater than or equal to 1

A constant variance model is fit

Total number of dose groups = 3

Total number of records with missing values = 0

Maximum number of iterations = 500

Relative Function Convergence has been set to: 1e-008; Parameter Convergence has been set to: 1e-00

Default Initial Parameter Values: alpha = 3.69; rho = 0 Specified; control = 5.5; slope = 9.72952e-028; power = -9999

Asymptotic Correlation Matrix of Parameter Estimates: ( *** The model parameter(s) -rho have been estimated at a boundary point, or have been specified by the user, and do not appear in the correlation matrix )

alpha control slope power

alpha 1 2.5e-007 4.7e-006 -4.6e-006

control 1.7e-008 1 -0.68 0.68

slope -5.3e-008 -0.68 1 -1

power 5.3e-008 0.68 -1 1

Parameter Estimates: 95.0% Wald Confidence Interval

Variable Estimate Std. Err. Lower Conf. Limit Upper Conf. Limit

alpha 3.28 0.892703 1.53033 5.02967

control 5.5 0.603963 4.31625 6.68375

slope 9.72954e-028 1.00134e-025 -1.95286e-025 1.97232e-025

power 15.1192 24.6187 -33.1325 63.3709

Table of Data and Estimated Values of Interest

Dose N Obs Mean Est Mean Obs Std Dev Est Std Dev Scaled Res.

------ --- -------- -------- ----------- ----------- ----------

22 9 5.5 5.5 1.5 1.81 -6.29e-008

55 9 5.7 5.7 2.1 1.81 2.02e-007

65 9 8 8 2.1 1.81 -6.02e-008

Degrees of freedom for Test A3 vs fitted <= 0

Model Descriptions for likelihoods calculated

Model A1: Yij = Mu(i) + e(ij)

Var{e(ij)} = Sigma^2

Model A2: Yij = Mu(i) + e(ij)

Var{e(ij)} = Sigma(i)^2

Model A3: Yij = Mu(i) + e(ij)

Var{e(ij)} = Sigma^2

Model A3 uses any fixed variance parameters thatwere specified by the user

Model R: Yi = Mu + e(i)

Var{e(i)} = Sigma^2

Likelihoods of Interest

Model Log(likelihood) # Param's AIC

A1 -29.535886 4 67.071772

A2 -28.913987 6 69.827974

A3 -29.535886 4 67.071772

fitted -29.535886 4 67.071772

R -34.003578 2 72.007156

Explanation of Tests

Test 1: Do responses and/or variances differ among Dose levels?

(A2 vs. R)

Test 2: Are Variances Homogeneous? (A1 vs A2)

Test 3: Are variances adequately modeled? (A2 vs. A3)

Test 4: Does the Model for the Mean Fit? (A3 vs. fitted)

(Note: When rho=0 the results of Test 3 and Test 2 will be the same.)

Tests of Interest

Test -2*log(Likelihood Ratio) Test df p-value

Test 1 10.1792 4 0.03752

Test 2 1.2438 2 0.5369

Test 3 1.2438 2 0.5369

Test 4 1.49214e-013 0 NA

The p-value for Test 1 is less than .05. There appears to be a difference between response and/or variances among the dose levels

It seems appropriate to model the data. The p-value for Test 2 is greater than .1. A homogeneous variance

model appears to be appropriate here

The p-value for Test 3 is greater than .1. The modeled variance appears to be appropriate here

NA - Degrees of freedom for Test 4 are less than or equal to 0. The Chi-Square

test for fit is not valid

Benchmark Dose Computation

Specified effect = 1

Risk Type = Estimated standard deviations from the control mean

Confidence level = 0.95

BMD = 63.6288

BMDL = 56.3188

**Supplementary Data S2. BMD modelling for mean volume of tumours (mm^3^) from Li et al. (2003) [34]: raw data from Table II, groups 4,5,6. The temperature for the control group was not stated and assumed as being 22°C (average room temperature for testing of chemicals according to OECD guideline).**

====================================================================

Power Model. (Version: 2.18; Date: 05/19/2014)

BMDS Model Run

~~~~~~~~~~~~~~~~~~~~~~~~~~~~~~~~~~~~~~~~~~~~~~~~~~~~~~~~~~~~~~~~~~~~~

The form of the response function is:

Y[dose] = control + slope * dose^power

Dependent variable = Mean_volume

Independent variable = Temperature

rho is set to 0

The power is restricted to be greater than or equal to 1

A constant variance model is fit

Total number of dose groups = 3

Total number of records with missing values = 0

Maximum number of iterations = 500

Relative Function Convergence has been set to: 1e-008

Parameter Convergence has been set to: 1e-008

Default Initial Parameter Values: alpha = 456.247; rho = 0 Specified; control = 39.1; slope = 9.25939e-009; power = -9999

Asymptotic Correlation Matrix of Parameter Estimates

( *** The model parameter(s) -rho

have been estimated at a boundary point, or have been specified by the user,

and do not appear in the correlation matrix )

alpha control slope power

alpha 1 1.7e-008 1.5e-006 -4.5e-007

control 3.7e-009 1 -0.54 0.52

slope -1.3e-008 -0.54 1 -1

power 1.3e-008 0.52 -1 1

Parameter Estimates

95.0% Wald Confidence Interval

Variable Estimate Std. Err. Lower Conf. Limit Upper Conf. Limit

alpha 405.553 110.377 189.217 621.888

control 39.0108 6.98265 25.325 52.6965

slope 1.05684e-008 2.24184e-007 -4.28823e-007 4.4996e-007

power 5.15976 5.067 -4.77139 15.0909

Table of Data and Estimated Values of Interest

Dose N Obs Mean Est Mean Obs Std Dev Est Std Dev Scaled Res.

------ --- -------- -------- ----------- ----------- ----------

22 9 39.1 39.1 24.9 20.1 -1.05e-008

55 9 49.1 49.1 17.3 20.1 5.52e-008

65 9 62.9 62.9 21.2 20.1 3.71e-009

Degrees of freedom for Test A3 vs fitted <= 0

Model Descriptions for likelihoods calculated

Model A1: Yij = Mu(i) + e(ij)

Var{e(ij)} = Sigma^2

Model A2: Yij = Mu(i) + e(ij)

Var{e(ij)} = Sigma(i)^2

Model A3: Yij = Mu(i) + e(ij)

Var{e(ij)} = Sigma^2

Model A3 uses any fixed variance parameters that

were specified by the user

Model R: Yi = Mu + e(i)

Var{e(i)} = Sigma^2

Likelihoods of Interest

Model Log(likelihood) # Param's AIC

A1 -94.570883 4 197.141765

A2 -93.986108 6 199.972217

A3 -94.570883 4 197.141765

fitted -94.570883 4 197.141765

R -97.417754 2 198.835507

Explanation of Tests

Test 1: Do responses and/or variances differ among Dose levels?

(A2 vs. R)

Test 2: Are Variances Homogeneous? (A1 vs A2)

Test 3: Are variances adequately modeled? (A2 vs. A3)

Test 4: Does the Model for the Mean Fit? (A3 vs. fitted)

(Note: When rho=0 the results of Test 3 and Test 2 will be the same.)

Tests of Interest

Test -2*log(Likelihood Ratio) Test df p-value

Test 1 6.86329 4 0.1433

Test 2 1.16955 2 0.5572

Test 3 1.16955 2 0.5572

Test 4 0 0 NA

The p-value for Test 1 is greater than .05. There may not be a

diffence between responses and/or variances among the dose levels

Modelling the data with a dose/response curve may not be appropriate

The p-value for Test 2 is greater than .1. A homogeneous variance model appears to be appropriate here

The p-value for Test 3 is greater than .1. The modeled variance appears to be appropriate here

NA - Degrees of freedom for Test 4 are less than or equal to 0. The Chi-Square

test for fit is not valid

Benchmark Dose Computation

Specified effect = 1

Risk Type = Estimated standard deviations from the control mean

Confidence level = 0.95

BMD = 62.8835

BMDL = 25.8432
